# Leisure-time physical activity and metabolomic trajectories over the transition from late midlife to old age

**DOI:** 10.1093/gerona/glag159

**Published:** 2026-06-11

**Authors:** Katri Ruutu, Jens Verbeeren, Tuija M Mikkola, Merja K Laine, Johan G Eriksson, Niko S Wasenius

**Affiliations:** Faculty of Medicine, University of Helsinki, Helsinki, Finland; Folkhälsan Research Center, Helsinki, Finland; Folkhälsan Research Center, Helsinki, Finland; Folkhälsan Research Center, Helsinki, Finland; Clinicum, Faculty of Medicine, University of Helsinki, Helsinki, Finland; Folkhälsan Research Center, Helsinki, Finland; Department of General Practice and Primary Health Care, University of Helsinki and Helsinki University Hospital, Helsinki, Finland; Folkhälsan Research Center, Helsinki, Finland; Department of General Practice and Primary Health Care, University of Helsinki and Helsinki University Hospital, Helsinki, Finland; Institute for Human Development and Potential, Agency for Science, Technology and Research, Singapore, Singapore; Yong Loo Lin School of Medicine, National University of Singapore, Singapore, Singapore; Folkhälsan Research Center, Helsinki, Finland; Department of General Practice and Primary Health Care, University of Helsinki and Helsinki University Hospital, Helsinki, Finland; (Biological Sciences Section)

**Keywords:** Biological aging, Metabolic aging, Metabolomics, Leisure-time physical activity

## Abstract

Physical activity may counteract aging-associated disruptions in the metabolome. We investigated how leisure-time physical activity (LTPA) associates with the metabolome in late midlife and with 15-year metabolomic trajectories. The study included 1816 Helsinki Birth Cohort Study participants at baseline (mean age 61.6 years), with 985, 996, and 740 participants at the 5-, 10-, and 15-year follow-ups, respectively. LTPA was assessed at baseline and after 15 years using a validated questionnaire and expressed as METhours/week. Nuclear magnetic resonance-based metabolomics data from fasting blood samples were used to analyze 161 biomarkers at baseline and at each follow-up. Weighted Gene Co-Expression Network Analysis was performed on the metabolomics data to identify 11 metabolomic biomarker modules. Baseline associations were analyzed using restricted cubic splines and longitudinal associations with linear mixed-effects models. Analyses were sex-stratified, covariate-adjusted, and corrected for multiple testing. At baseline, LTPA was nonlinearly associated with albumin in women and with 9 biomarkers in men in the crude, but not fully adjusted, model. Over the follow-up, baseline LTPA was associated with the age-related trajectory of triglyceride-enriched high-density lipoprotein (HDL) module in men (age × LTPA interaction *p* = .041). In the high LTPA group, the trajectory declined significantly compared to the low (*p* = .004) and moderate LTPA groups (*p* = .045). No associations were found in women. To conclude, in men, higher LTPA in late midlife predicted a favorable 15-year trajectory of triglyceride-enriched HDL. Our findings indicate that an active lifestyle is associated with more favorable metabolomic trajectories related to cardiometabolic health across late midlife and old age.

## Introduction

Biological aging is a progressive, multifactorial process characterized by molecular and cellular dysregulation across multiple biological systems.^1^ Systemic metabolic alterations, collectively referred to as metabolic aging, represent a key manifestation of this process.^2^^,^^3^ Several hallmarks of aging, including deregulated nutrient sensing, mitochondrial dysfunction, and chronic low-grade inflammation, are closely linked to unfavorable age-related shifts in the ensemble of small-molecule metabolites in biological systems, called the metabolome.^1^^,^^3^ Metabolomics, the comprehensive profiling of the metabolome, provides functional insights into these processes and serves as a sensitive tool for assessing biological aging and metabolic health.^4^^,^^5^

Physical activity is associated with favorable changes in the metabolome,^6^ and emerging evidence suggests that it may also be related to slower metabolic aging.^7^^,^^8^ Observational metabolomics studies have linked higher physical activity levels to favorable lipid profiles,^9–12^ carbohydrate^10^^,^^12^^,^^13^ and amino acid metabolites,^10^^,^^13–17^ as well as reduced inflammatory markers.^12^^,^^16^ However, these studies are exclusively cross-sectional, limiting insights into temporal dynamics.^18^ Moreover, differences in metabolite coverage, participant age, sample sizes, and physical activity assessment methods constrain comparability and generalizability.

Physical activity is a multidimensional behavior encompassing occupational, commuting, and leisure-time activities.^19^ Among these domains, leisure-time physical activity (LTPA), including exercise and recreational pursuits, is particularly relevant in late midlife and older adulthood because it is largely voluntary, potentially modifiable,^19^ and directly targeted by physical activity guidelines and lifestyle interventions.^20^ Moreover, because LTPA often increases around the retirement transition, whereas total physical activity may not, LTPA may represent an increasingly important and modifiable contributor to habitual physical activity and cardiometabolic health in this life stage.^21^^,^^22^ To date, only a few studies have examined LTPA in relation to metabolic aging based on composite metabolomics-based aging markers,^7^^,^^8^ and longitudinal evidence remains particularly limited. These studies suggest that higher volumes of LTPA^7^ and adherence to physical activity guidelines^8^ are associated with more favorable metabolomics-based aging. However, previous work has focused on composite metabolomics-based aging markers rather than long-term trajectories of individual metabolites or metabolite modules representing shared biological pathways. Thus, further longitudinal metabolome-wide studies are needed to clarify how LTPA may shape metabolic aging trajectories in late midlife and old age.

To address this gap, we investigated associations between LTPA and metabolomic profiles over 15 years in the Helsinki Birth Cohort Study (HBCS), a population-based cohort of older adults. Specifically, we examined cross-sectional associations between LTPA and the metabolome in late midlife, and longitudinal metabolomic trajectories across varying baseline LTPA levels and changes in LTPA over the 15-year follow-up.

## Methods

### Study design and participants

**Figure 1:**
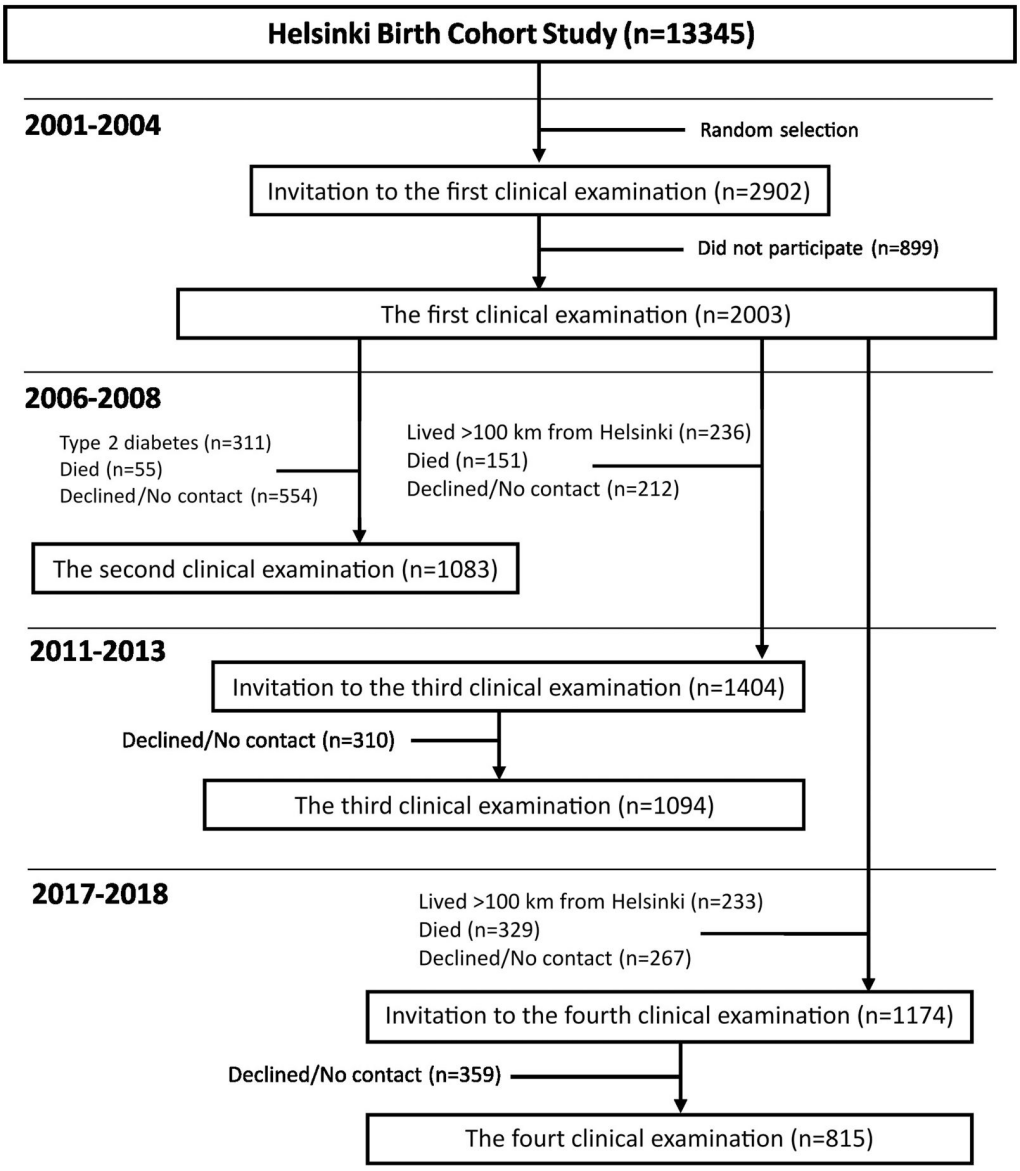
The study design of the Helsinki Birth Cohort Study.

The study participants of this prospective cohort study are part of HBCS (Figure 1), which comprised 13 345 individuals born between 1934 and 1944 at either the Helsinki University Central Hospital or the Helsinki City Maternity Hospital, and who, during that period, attended child welfare clinics in the city. From this initial group, 8760 individuals born at the Helsinki University Hospital and still alive in 1971, when Finland introduced a unique personal identification number for its residents, were identified. From this subset, 2902 individuals who were alive and residing in Finland were randomly selected using random-number tables and invited to participate in the first clinical examination conducted between 2001 and 2004. Of those invited, 2003 agreed to take part. A second clinical examination took place in 2006-2008, targeting participants who were still alive and had not been diagnosed with diabetes at the time of the first assessment. This round included 1083 individuals. The third clinical follow-up was performed in 2011-2013, and 1404 participants, who lived within 100 km of the study clinic in Helsinki and were still alive, were invited. Of those invited, 1094 participants took part. The fourth clinical follow-up, conducted between 2017 and 2018, included participants who were still alive and living within a 100-kilometer radius of the study clinic in Helsinki. A total of 1174 individuals were invited, and ultimately, 815 attended the fourth clinical examination.

In this study, we used data from each clinical examination. The baseline sample of this study consisted of 1816 participants (90.7% of 2003 attendees) after excluding those without complete information on covariates, LTPA, or metabolomics at baseline. At the follow-up visits, participants were included if they were part of the baseline analytical sample and provided a blood sample with usable metabolomics data: 91.0%, 91.0%, and 90.8% of clinical examination attendees were included in the analytical sample at the second, third, and fourth visits, respectively. Thus, the analytical samples at the second, third, and fourth clinical examinations consisted of 985, 996, and 740 individuals, respectively, with a total of 4537 observations.

The study received approval from both the Ethics Committee of the Hospital District of Helsinki and Uusimaa and the Ethics Committee of the National Public Health Institute, Helsinki. All participants provided written informed consent prior to undergoing any study-related procedures.

### Leisure-time physical activity

We evaluated LTPA of each participant on two occasions: initially at baseline during the first clinical examination conducted between 2001 and 2004, and again during the fourth clinical assessment in 2017-2018. We determined LTPA using a validated 12-month recall questionnaire from the Kuopio Ischemic Heart Disease (KIHD) study,^23^^,^^24^ which is a modified version of the Minnesota leisure-time activity questionnaire. In the KIHD questionnaire, participants reported all types of physical activities they had engaged in over the past year, such as cycling, walking, household chores, and gardening. They were also given the option to list any additional activities not included in the examples. For each activity reported, participants indicated its intensity (ranging from 0 = recreational, to 1 = conditioning, 2 = brisk conditioning, and 3 = competitive, strenuous exercise), frequency (times per month), and average duration. We then assigned a metabolic equivalent of task (MET) value to each activity based on its reported intensity, using the established MET databases.^25^ The MET value reflects the absolute intensity of the activity, defined as the ratio of the metabolic rate during the activity to the resting metabolic rate (1 MET = 3.5 mL O_2_ · kg^−1^ · min^−1^ or 1 kcal · kg^−1^ · h^−1^). The volume of each activity (MET-hours) was calculated by multiplying its MET value by the average duration and frequency. Finally, we summed the volumes of all reported activities to determine the total volume of LTPA, expressed in hours of metabolic equivalent of task per week (METh/week).

In the analyses, we utilized LTPA both as a continuous variable and a categorical variable. LTPA was divided into 3 categories: (1) Low: less than 16.7 METh/week, (2) Moderate: 16.7 to less than 50 METh/week, and (3) High: 50 METh/week or more. The thresholds between the categories are comparable to the current physical activity recommendations^20^ as the first LTPA threshold 16.7 METh/week (equivalent to 1000 METmin/week) is twice the minimum recommended level of physical activity, and the second threshold 50 METh/week (=3000 METmin/week) is 4 times the minimum recommended amount in the current guidelines.

### Metabolomics

For the metabolomics analysis, we collected venous blood samples from participants after a 12-h overnight fast at each of the 4 clinical examinations. Participants were also instructed to avoid strenuous physical activity on the day before and on the morning of blood sampling. We used a high-throughput nuclear magnetic resonance (NMR) metabolomics platform for the simultaneous quantification of 250 metabolic biomarkers (Nightingale Health Ltd., Helsinki, Finland). Biomarker types included were amino acids, glycolysis-related metabolites, ketone bodies, inflammation, fluid balance, cholesterol, triglycerides, phospholipids, cholesteryl esters, free cholesterol, total lipids, lipoprotein particle concentration and size, other lipids, apolipoproteins, fatty acids and their ratios, and 14 lipoprotein subclasses. The used automated method provides absolute concentrations of the metabolites with high reproducibility and enables the measurement of lipoprotein particles of different sizes, in different concentrations, and their components. The details of the experimentation have been previously described.^26–28^

To mitigate batch effects between the clinical examinations, we utilized a previously reported algorithm,^29^ using the latest visit (2017-2018) as the reference. For each sequential time interval, 500 age- and sex-matched individuals (250 men and 250 women) were selected to correct the levels of the metabolomic biomarkers. Age and sex were chosen as matching criteria due to their known influence on metabolite profiles, with mean ages of 74.5 and 74.0 years (for the 2017-2018 to 2011-2013 correction), 68.7 and 68.5 years (for the 2011-2013 to 2006-2008 correction), and 63.9 and 64.0 years (for the 2006-2008 to 2001-2004 correction). Metabolomic biomarkers expressed as ratios or percentages were recalculated after the batch correction.

Metabolomic biomarkers with 20% or more missing values, below quality thresholds, or values below detection limits were excluded, resulting in the removal of 15 metabolomic biomarkers (Table S1). To avoid redundancy and focus on absolute concentrations, we excluded ratios and relative (%) lipoprotein measures, resulting in 161 metabolomic biomarkers for analysis (Table S1). Values below the detection limit were imputed using random values drawn from a uniform distribution between the lowest observed value for each metabolomic biomarker and half of that value. Across all observations from 1890 participants with complete baseline metabolomics data, corresponding to 4700 observations across 4 timepoints, 0.37% of values across the 161 retained biomarkers required imputation (Table S1). In addition, metabolomic biomarker values exceeding 5 standard deviations from the mean were excluded from analysis to mitigate outlier influence. Outlier exclusion affected 139 of 161 metabolomic biomarkers and removed 800 data points (0.11%); per-biomarker outlier counts are provided in Table S1. Finally, metabolomic biomarker values were log-transformed to reduce skewness and z-scaled to standardize the data for analysis.

### Weighted gene co-expression network analysis

To identify metabolomic biomarker modules representing shared biological pathways, we performed Weighted Gene Co-expression Network Analysis (WGCNA) on the metabolomics data using the WGCNA R package.^30^ We used cross-sectional data from the 2001-2004 visit, which served as the baseline of our longitudinal cohort and provided the largest sample size, including 1890 participants with complete metabolomic biomarker data and 161 metabolomic biomarkers. Men and women were analyzed together to maximize statistical power for module detection and to identify metabolomic networks common to both sexes, resulting in 11 modules (Table S2): (1) Cholesterol-rich, apolipoprotein B (ApoB) containing low-density lipoprotein (LDL) and intermediate-density lipoprotein (IDL) particle module (referred to as LDL-ApoB module), (2) Large triglyceride-rich very-low-density lipoprotein (VLDL) particle module (large VLDL module), (3) General and medium apolipoprotein A1 (ApoA1) containing high-density lipoprotein (HDL) particle module (HDL-ApoA1 module), (4) General and small VLDL particle module (small VLDL module), (5) Large HDL particle module (large HDL module), (6) Triglyceride-enriched lipoproteins and fatty acids module (TG-FA module), (7) Branched-chain amino acids module (BCAA module), (8) Phospholipid and choline module (phospholipid module), (9) Extra-small VLDL particle module (extra-small VLDL module), (10) Small HDL particle module (small HDL module), and (11) Triglyceride-enriched HDL module (HDL-TG module). The following 22 metabolomic biomarkers were not assigned to any specific module and thus they were used as such in the analyses: LDL size, degree of fatty acid unsaturation, omega-3 fatty acids, docosahexaenoic acid, alanine, glutamine, glycine, histidine, phenylalanine, tyrosine, glucose, lactate, pyruvate, citrate, glycerol, 3-hydroxybutyrate, acetate, acetoacetate, acetone, creatinine, albumin, and glycoprotein acetyls (GlycA). To assess the robustness of the combined-sex module derivation, WGCNA was repeated separately in men (*n* = 884) and women (*n* = 1006) using identical parameters and complete metabolomics data. Both sex-stratified networks yielded comparable module structures, with 11 and 12 modules in men and women, respectively. Module membership across all 3 networks is provided in Table S3.

In WGCNA, network construction used Spearman correlations transformed for a signed network approach ((1 + *r*)/2). A soft-thresholding power (*β*) of 20 (*R*^2^ = 0.567) was selected to maximize scale-free topology fit while maintaining adequate mean connectivity. The adjacency matrix was transformed into a topological overlap matrix (TOM). Module detection used hierarchical clustering (Ward’s method) on TOM dissimilarity, followed by dynamic tree cutting (minimum module size = 3, deep split = 3). Module eigengenes were calculated as the first principal component of standardized (z-scaled, mean = 0, standard deviation (SD) = 1) metabolomic biomarker values within each module. Modules with eigengene correlations >0.85 were merged. Metabolomic biomarkers were reassigned between modules if module membership (kME) with a new module exceeded 0.80 and was ≥20% greater than with the original module. Metabolomic biomarkers assigned to the grey module (unassigned) were analyzed individually rather than as module eigengenes.

Module membership was fixed based on baseline WGCNA results. For the cross-sectional analysis, module eigengenes were calculated separately for men and women and standardized to facilitate interpretation in SD units. For the longitudinal analysis, sex-stratified eigengenes were calculated using principal component analysis applied to standardized metabolomic biomarker values pooled from all available timepoints, thereby preserving temporal variation, and were then standardized.

### Covariates

Covariate information for this study was gathered at baseline during the participants’ initial clinical assessment. Height was measured to the nearest 0.1 cm using KaWe stadiometers, and body weight was recorded to the nearest 0.1 kg with SECA alpha 770 medical scales while participants wore light indoor clothing. Body mass index (BMI, kg/m^2^) was calculated by dividing weight in kilograms by the square of height in meters.

A standardized oral glucose tolerance test (OGTT), involving the ingestion of 75 grams of glucose over 2 hours, was used to assess glucose (mmol/L) and insulin (mU/L) levels at fasting, 30 minutes, and 120 minutes. This test also served to identify potential undiagnosed diabetes. Diagnostic criteria from the World Health Organization (WHO)^31^ were applied, where diabetes is defined by a fasting plasma glucose level of ≥7.0 mmol/L or a 2-hour plasma glucose level of ≥11.1 mmol/L during the OGTT. Data on previously diagnosed diabetes were obtained through self-reported information, national registers, or evidence of diabetes/glucose-lowering medication use.

The participants completed standardized questionnaires assessing various aspects of their health and lifestyle. Smoking status was categorized into 4 groups: (1) never smoked, (2) quit smoking (former smoker), (3) occasional smoking, and (4) regular/daily smoking. Alcohol consumption was classified as follows: (1) never or former drinkers, (2) light/monthly consumption (1-2 times per month), and (3) regular/weekly or more frequent consumption (1-2 times per week). The participants reported the presence of chronic diseases and conditions, including cardiovascular diseases and conditions (congestive heart failure, hypertension, intermittent claudication, angina pectoris, previous myocardial infarction, and stroke), pulmonary diseases (asthma, emphysema, and chronic bronchitis), musculoskeletal disorders (osteoporosis), and cancer. Based on these reports, the number of chronic diseases and conditions was categorized as follows: 0 = no chronic diseases or conditions, 1 = one chronic disease or condition, and 2 = two or more chronic diseases or conditions. Educational attainment was used as a proxy for socioeconomic status and classified into 1 of 4 groups: (1) primary or less, (2) upper secondary, (3) lower tertiary, and (4) upper tertiary.

Dietary intake information was calculated as previously described.^32^ Briefly, at baseline (years 2001-2004), participants completed a validated, semi-quantitative 128-item food-frequency questionnaire (FFQ) covering 12 food groups. After quality assurance by a study nurse, data were processed at the National Institute for Health and Welfare with the use of the National Food Consumption Database FINELI (National Institute for Health and Welfare, Helsinki, Finland).^33^ Total energy intake (kJ/day) was calculated, and macronutrient composition was expressed as a percentage of total energy: total fat, fat subfractions (saturated fatty acids, monounsaturated fatty acids, polyunsaturated fatty acids, protein, carbohydrate, and alcohol).

### Statistical analysis

Baseline characteristics are reported as percentages for categorical variables and means with SDs for continuous variables. All analyses were sex-stratified due to the known metabolic differences between men and women.^34^^,^^35^

#### Cross-sectional analysis at baseline

We examined non-linear relationships between continuous LTPA and metabolomic modules and biomarkers at baseline. Eigengenes from WGCNA-detected modules were used as dependent variables in the analysis, while metabolomic biomarkers not assigned to any specific module were treated as individual metabolomic biomarkers in the analysis. This approach resulted in a reduction in data dimensionality, reducing multiple-testing burden and increasing statistical power. To address the right-skewed distribution of LTPA and enhance robustness, we applied 0.5% Winsorization.

To examine the non-linear relationships between continuous LTPA and the metabolomic biomarker modules or individual metabolomic biomarkers, restricted cubic splines (RCS) were used with two spline specifications: RCS1 with internal knots at the 10^th^ and 90^th^ percentiles (and linear extrapolation beyond) and an inner knot at 50^th^ percentile LTPA values yielding 2 degrees of freedom, and RCS2 with boundary knots at the 5^th^ and 95^th^ percentiles, and inner knots at 35^th^ and 65^th^ percentile LTPA values yielding 3 degrees of freedom. To determine optimal functional forms, we compared RCS1 and RCS2 models using tenfold cross-validation and selected the model with the lowest residual sum of squares for each module or metabolomic biomarker. After this, to evaluate the association between continuous LTPA and each module eigengene or metabolomic biomarker, we developed a crude model that was adjusted for age and a fully adjusted model that was adjusted for age, BMI, educational level, alcohol consumption, smoking, diabetes, chronic diseases, and dietary variables including total energy intake and the percentage of proteins, carbohydrates, polyunsaturated fatty acids, and monounsaturated fatty acids from total energy intake.

Associations between continuous LTPA and module eigengenes or metabolomic biomarkers were evaluated using permutation-based *p*-values (10 000 permutations), based on likelihood ratio (LR) test statistics comparing models with and without LTPA terms. False discovery rate (FDR) control was applied to permutation-based *p*-values using the Benjamini-Hochberg procedure and applied separately within each sex, across all module eigengenes and individual biomarkers simultaneously. FDR-corrected *p*-values are hereafter reported as *q*-values. Statistical significance was assessed using both nominal *p*-values (*p* < .05) and *q*-values (*q* < .05). Module or individual metabolomic biomarker curves with 95% confidence intervals (CI) were plotted using average adjusted predictions (AAP) and 1000 bootstrap samples across the full LTPA range.

#### Longitudinal analysis

Two LTPA exposures were examined in the longitudinal analysis. First, baseline LTPA was categorized as low, moderate, or high. Second, change in LTPA was assessed using standardized residuals from regressing the follow-up LTPA at the final visit in 2017-2018 on baseline LTPA and baseline age, computed separately by sex. Standardized residual change in LTPA was then categorized as decreased (the standardized residual change in LTPA ≤ −0.5 SD), stable (the standardized residual change in LTPA = −0.5 to 0.5 SD), or increased (the standardized residual change in LTPA ≥ 0.5 SD).

Linear mixed-effects models were used to examine whether metabolomic module eigengene and metabolomic biomarker trajectories with age differed between LTPA categories. The same model structure was applied separately for baseline LTPA categories and for LTPA change categories as the exposure of interest. Models included random intercepts and random slopes for age when convergence permitted; otherwise, random intercepts only. Metabolite trajectories were modeled using RCS for age (one internal knot at the median; boundary knots at the 10^th^ and 90^th^ percentiles). Age × LTPA interactions were tested using LR tests. When the interaction was nominally significant (*p* < .05), pairwise contrasts between LTPA categories were derived from Wald tests of the interaction coefficients using a dual-reference approach. To obtain all pairwise comparisons, models were refitted using different LTPA reference categories, first moderate and then low LTPA. FDR correction was applied separately within each sex stratum: first across all age × LTPA interaction *p*-values, and then across all pairwise contrast *p*-values pooled over all outcomes. Population-averaged trajectories were estimated using AAP, with 95% CIs from 250 bootstrap iterations. All models were adjusted for BMI and diabetes as time-varying covariates, and for the following baseline covariates: total energy intake, macronutrient composition (carbohydrate, protein, polyunsaturated fatty acids, and monounsaturated fatty acids), educational level, chronic disease burden, smoking status, and alcohol consumption.

All analyses were conducted using Python version 3.12.7 (Python Software Foundation, https://www.python.org/). Linear mixed-effects models were fitted using statsmodels (version 0.14.5). LR tests and CI calculations used scipy. Bootstrap resampling and permutation processing were parallelized using joblib. Module eigengenes were calculated using principal component analysis implemented in scikit-learn. RCS were implemented using the patsy library’s cr() function through the statsmodels formula interface. FDR correction was performed using the Benjamini-Hochberg procedure via statsmodels. Data manipulation was performed using pandas and numpy, and figures were generated using matplotlib and seaborn.

## Results

### Participants’ characteristics

Baseline characteristics of the participants are presented in Table 1 for women (*n* = 966) and in Table 2 for men (*n* = 850). At baseline, the mean age of the participants was 61.6 years (SD = 2.9). The follow-up visits took place at 4.9 years (SD = 0.8), 9.8 years (SD = 0.8), and 15.3 years (SD = 0.9) after the baseline. The corresponding mean ages of the participants at the follow-up visits were 66.4 years (SD = 2.8), 71.0 years (SD = 2.7), and 76.4 years (SD = 2.7), respectively. Participants who attended the final visit (completers) differed from those who did not (non-completers) with respect to age, BMI, diabetes, and chronic disease burden, educational attainment, smoking, and alcohol use both in women (Table S4) and in men (Table S5). Regarding LTPA, women showed no significant difference in LTPA category distribution (*p* = .055), although non-completers had higher continuous LTPA (48.8 vs 43.2 METh/week, *p* = .026), whereas men showed no differences in continuous LTPA (*p* = .440) but fewer completers in the lowest LTPA category (13.8% vs 23.7%, *p* = .002).

**Table 1:** Baseline characteristics of women across leisure-time physical activity categories.

Characteristics	Combined (*n* = 966)	Low LTPA (*n* = 195)	Moderate LTPA (*n* = 448)	High LTPA
				(*n* = 323)
**Age (years), mean (SD)**	61.6 (3.0)	61.0 (3.0)	61.5 (3.0)	62.1 (3.1)
**Body mass index (kg/m^2^), mean (SD)**	27.7 (5.0)	28.8 (5.5)	27.5 (5.1)	27.3 (4.5)
**Educational status, *n* (%)**				
Primary or less	371 (38.4)	76 (39.0)	165 (36.8)	130 (40.2)
Upper secondary	161 (16.7)	26 (13.3)	78 (17.4)	57 (17.6)
Lower tertiary	245 (25.4)	57 (29.2)	110 (24.6)	78 (24.1)
Upper tertiary	189 (19.6)	36 (18.5)	95 (21.2)	58 (18.0)
**Smoking, *n* (%)**				
Never	533 (55.2)	97 (49.7)	257 (57.4)	179 (55.4)
Former	236 (24.4)	47 (24.1)	100 (22.3)	89 (27.6)
Light	55 (5.7)	10 (5.1)	27 (6.0)	18 (5.6)
Regular	142 (14.7)	41 (21.0)	64 (14.3)	37 (11.5)
**Alcohol use, *n* (%)**				
None	67 (6.9)	16 (8.2)	26 (5.8)	25 (7.7)
Light	522 (54.0)	97 (49.7)	244 (54.5)	181 (56.0)
Regular	377 (39.0)	82 (42.1)	178 (39.7)	117 (36.2)
**Chronic diseases, *n* (%)**				
None	462 (47.8)	87 (44.6)	222 (49.6)	153 (47.4)
1 disease	347 (35.9)	76 (39.0)	152 (33.9)	119 (36.8)
≥2 diseases	157 (16.3)	32 (16.4)	74 (16.5)	51 (15.8)
**Diabetes status at baseline, yes *n* (%)**	129 (13.4)	31 (15.9)	58 (12.9)	40 (12.4)
**LTPA (METh/week), mean (SD)**	46.4 (39.4)	10.6 (4.2)	31.9 (9.2)	88.1 (41.4)
**Dietary intake, mean (SD)**				
Total energy (kJ/day)	8508 (2923)	8198 (2967)	8623 (3021)	8535 (2748)
Protein (E%)	17.4 (2.5)	17.4 (2.5)	17.4. (2.5)	17.3 (2.6)
Carbohydrates (E%)	47.8 (6.7)	47.8 (6.7)	47.6 (6.4)	48. (7.1)
Fat (E%)	33.1 (5.3)	32.9 (5.5)	33.2 (5.2)	33.2 (5.5)
Saturated fat (E%)	12.1 (2.6)	12.0 (2.7)	12.1 (2.5)	12.2 (2.6)
PUFA (E%)	5.3 (1.3)	5.3 (1.2)	5.3 (1.3)	5.3 (1.3)
MUFA (E%)	10.9 (2.1)	10.9 (2.1)	11.0 (2.1)	10.9 (2.2)
Alcohol (E%)	1.9 (2.7)	2.2 (3.4)	1.9 (2.5)	1.7 (2.5)

Abbreviations: LTPA, leisure-time physical activity; MUFA, monounsaturated fatty acids; PUFA, polyunsaturated fatty acids.

**Table 2:** Baseline characteristics of men across leisure-time physical activity categories.

Characteristics	Combined (*n* = 850)	Low LTPA (*n* = 169)	Moderate LTPA (*n* = 400)	High LTPA
				(*n* = 281)
**Age (years), mean (SD)**	61.5 (2.8)	60.9 (2.5)	61.5 (2.9)	61.9 (2.7)
**Body mass index (kg/m^2^), mean (SD)**	27.4 (4.0)	28.1 (4.6)	27.5 (3.8)	27.0 (3.8)
**Educational status, *n* (%)**				
Primary or less	222 (26.1)	47 (27.8)	102 (25.5)	73 (26.0)
Upper secondary	195 (22.9)	45 (26.6)	86 (21.5)	64 (22.8)
Lower tertiary	233 (27.4)	54 (32.0)	117 (29.2)	62 (22.1)
Upper tertiary	200 (23.5)	23 (13.6)	95 (23.8)	82 (29.2)
**Smoking, *n* (%)**				
Never	226 (26.6)	28 (16.6)	121 (30.2)	77 (27.4)
Former	396 (46.6)	74 (43.8)	185 (46.2)	137 (48.8)
Light	51 (6.0)	7 (4.1)	30 (7.5)	14 (5.0)
Regular	177 (20.8)	60 (35.5)	64 (16.0)	53 (18.9)
**Alcohol use, *n* (%)**				
None	63 (7.4)	11 (6.5)	28 (7.0)	24 (8.5)
Light	225 (26.5)	32 (18.9)	109 (27.2)	84 (29.9)
Regular	562 (66.1)	126 (74.6)	263 (65.8)	173 (61.6)
**Chronic diseases, *n* (%)**				
None	420 (49.4)	73 (43.2)	213 (53.2)	134 (47.7)
1 disease	283 (33.3)	66 (39.1)	123 (30.8)	94 (33.5)
≥2 diseases	147 (17.3)	30 (17.8)	64 (16.0)	53 (18.9)
**Diabetes status at baseline, yes *n* (%)**	158 (18.6)	43 (25.4)	79 (19.8)	36 (12.8)
**LTPA (METh/week), mean (SD)**	45.5 (38.9)	9.1 (4.4)	32.0 (9.4)	86.8 (41.0)
**Dietary intake, mean (SD)**				
Total energy (kJ/day)	10 470 (3655)	10 270 (3982)	10 151 (3441)	11 044 (3689)
Protein (E%)	16.7 (2.5)	16.5 (2.9)	16.8 (2.6)	16.6 (2.3)
Carbohydrates (E%)	45.6 (6.6)	44.2 (6.9)	45.3 (6.4)	46.7 (6.7)
Fat (E%)	33.7 (5.6)	34.1 (6.4)	33.9 (5.4)	33.3 (5.2)
Saturated fat (E%)	12.1 (2.5)	12.3 (2.7)	12.2 (2.6)	12.0 (2.3)
PUFA (E%)	5.3 (1.2)	5.3 (1.3)	5.4 (1.2)	5.2 (1.1)
MUFA (E%)	11.3 (2.2)	11.5 (2.5)	11.4 (2.1)	11.2 (2.1)
Alcohol (E%)	4.3 (5.0)	5.6 (6.8)	4.3 (4.8)	3.6 (3.8)

Abbreviations: LTPA, leisure-time physical activity; MUFA, monounsaturated fatty acids; PUFA, polyunsaturated fatty acids.

### LTPA and baseline metabolomic biomarkers

At baseline, we observed nominally significant associations between LTPA and 5 biomarkers in women in the crude model: alanine (*p* = .011), citrate (*p* = .026), acetate (*p* = .037), albumin (*p* = .001), and GlycA (*p* = .009) (Table S6). Of these, 3 remained significant after covariate adjustment: alanine (*p* = .023), citrate (*p* = .021), and albumin (*p* = .006). In men, LTPA was nominally associated with 12 biomarkers in the crude model: large VLDL module (*p* = .023), small VLDL module (*p* = .037), extra-small VLDL module (*p* = .034), triglyceride-enriched lipoproteins and fatty acids module (*p* = .007), degree of fatty acid unsaturation (*p* = .006), alanine (*p* = .011), phenylalanine (*p* = .004), glucose (*p* = .009), lactate (*p* = .004), pyruvate (*p* = .001), glycerol (*p* < .001), and GlycA (*p* < .001) (Table S6). In the fully adjusted model, the association of LTPA was significant with two biomarkers: HDL-ApoA1 module (*p* = .009), and small HDL module (*p* = .007).

After FDR correction, significant associations between LTPA and metabolomic biomarkers were observed only in the crude model (Table S7). In women, LTPA was nonlinearly associated with albumin (*q* = .040) (Figure S1). In men, LTPA showed nonlinear associations with 9 metabolomic biomarkers (Figure S2): alanine (*q* = .041), phenylalanine (*q* = .030), glucose (*q* = .038), lactate (*q* = .030), pyruvate (*q* = .010), glycerol (*q* = .003), GlycA (*q* = .003), fatty acid unsaturation (*q* = .033), and triglyceride-enriched lipoproteins and fatty acids module (*q* = .033). In the fully adjusted model, no associations were found either in women or in men (Table S7).

### LTPA and longitudinal metabolomic trajectories

**Figure 2:**
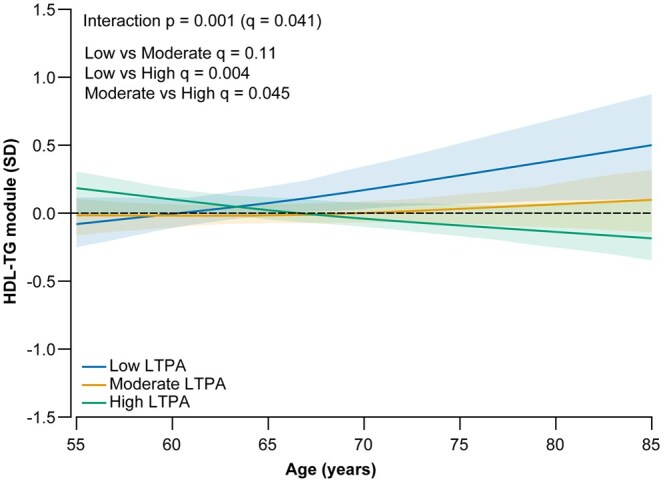
The age-related trajectories of the triglyceride-enriched high-density lipoprotein particle module (HDL-TG module) in the low (<16.7 METh/week), moderate (16.7 to <50 METh/week), and high (≥50 METh/week) leisure-time physical activity (LTPA) categories in men over the 15-year follow-up. *p*-value, the uncorrected significance level; *q*-value, the significance level after correcting for multiple testing.

In men, baseline LTPA was nominally associated with age-related trajectories of 4 metabolomic modules: large VLDL particle module (age × LTPA interaction *p* = .006), triglyceride-enriched lipoproteins and fatty acids module (age × LTPA interaction *p* = .034), branched-chain amino acids module (age × LTPA interaction *p* = .017), and triglyceride-enriched HDL module (age × LTPA interaction *p* = .001) (Table S8). Of these, the association between LTPA and triglyceride-enriched HDL module remained significant after correction for multiple testing (age × LTPA interaction *q* = .041). The age-related trajectory of triglyceride-enriched HDL module increased in the low and moderate LTPA groups and declined in the high LTPA group (Figure 2). Significant differences were observed between high versus low (*q* = .004) and high versus moderate LTPA groups (*q* = .045), but not between moderate versus low LTPA groups (*q* = .110) (Table S9). In women, we detected no significant age × LTPA interactions (Table S8).

### Long-term change in LTPA and longitudinal metabolomic trajectories

In women, the standardized residual change in LTPA was nominally associated with the age-related trajectories of the branched-chain amino acids module, histidine, and creatinine, but none of them remained significant after correction for multiple testing (Table S10). In men, LTPA change was not associated with any metabolomic trajectory (Table S10).

## Discussion

In this 15-year prospective cohort study, the key finding was that higher volumes of LTPA were associated with a more favorable age-related trajectory in a triglyceride-enriched HDL module among men. This observational finding is consistent with the possibility that physical activity accompanies more advantageous patterns of lipid metabolism during aging. The nonlinear associations observed between LTPA and individual metabolites in late midlife further suggest that physical activity may be linked to metabolomic profiles indicative of healthier metabolic function and aging. However, these associations should be interpreted cautiously, given the potential residual confounding and the interrelated nature of the covariates.

In our cross-sectional analyses in late midlife, we observed nonlinear associations of LTPA with lipid, amino acid, energy, and inflammatory metabolites in the crude model, but these associations attenuated after adjusting for covariates. Previous metabolomics and physical activity studies have reported comparable findings linking physical activity to lower systemic inflammation,^6^^,^^12^^,^^16^ improved energy metabolism,^6^^,^^10^^,^^12^^,^^13^ and favorable lipid profiles.^6^^,^^9–12^ However, this study extends prior evidence by modeling nonlinear associations and applying rigorous adjustment for multiple confounders. In this context, higher LTPA was associated with a more favorable metabolomic profile, suggesting potential relevance for cardiometabolic health and age-related metabolic processes. However, these findings should be interpreted cautiously, given the cross-sectional design and attenuation of associations after covariate adjustment. In addition, the most pronounced differences appeared to occur across the low-to-moderate LTPA range (≤50 METh/week), with no clear evidence of additional benefits at higher LTPA volumes, although estimates at very high LTPA levels were less precise. This pattern is broadly consistent with prior dose-response evidence between physical activity and health outcomes^36–38^ and with WHO’s physical activity guidelines.^20^ Nonetheless, the interplay between LTPA and closely related covariates such as BMI, diet, smoking, alcohol intake, and chronic diseases on metabolomic biomarkers should be carefully studied.

Our longitudinal findings indicated that triglyceride-enriched HDL particles declined with age among men with high LTPA and increased in those with low or moderate volumes of LTPA. This pattern suggests that higher LTPA in late midlife may attenuate or reverse the age-related increase in HDL particle triglyceride content, a feature linked to cardiometabolic risk.^39^ Moderate LTPA may also confer partial protection, whereas low levels of LTPA may potentially result in worsening lipid metabolism. Our finding is consistent with previous studies showing that aging is associated with reduced clearance of triglycerides and alterations in triglyceride metabolism^40^ and that physical activity may attenuate age-related dyslipidemia by enhancing triglyceride clearance and HDL functionality.^41–43^ Furthermore, nominal associations between higher LTPA and more favorable trajectories of 3 triglyceride-related modules–large VLDL particle module, triglyceride-enriched lipoproteins and fatty acids module, and branched-chain amino acids module–may suggest broader improvements in triglyceride-rich lipoprotein metabolism but require cautious interpretation and further study. Notably, the lower limit of the high LTPA group was broadly comparable to approximately 4 times the current minimum physical activity recommendation.^20^ While the greatest relative health gains are generally observed when moving from inactivity to some activity, additional physical activity may confer further benefits.^20^^,^^44^ Accordingly, the more favorable long-term triglyceride-related metabolic trajectories observed among men with high LTPA may be clinically relevant for cardiometabolic risk reduction, while not implying that such benefits are restricted to very high activity volumes. This comparison should nevertheless be interpreted with some caution, as the KIHD questionnaire captures a broad range of leisure-time activities and may thus yield relatively high LTPA estimates. However, these estimates remain broadly comparable with the questionnaire-based evidence underpinning the current physical activity guidelines.

Lack of associations between the change in LTPA over 15 years and age-related metabolomic trajectories suggests that physical activity changes from late midlife to old age may exert no, modest, or only transient effects on individual metabolites and systemic metabolism. This finding extends our previous findings,^7^ where an increase in the volume of LTPA was associated with a lower metabolomics-based biological age, indicating decelerated metabolic aging. Together, these findings imply that composite metabolomics-based aging measures may better capture the effects of physical activity during aging.

The observed attenuation of associations between LTPA and metabolomic biomarkers after full covariate adjustment suggests confounding by BMI, diet, and other correlated health behaviors.^45^ Crude associations may therefore reflect broader lifestyle patterns rather than direct metabolic effects of LTPA. This attenuation of associations could indicate confounding or partial mediation by sociodemographic and behavioral factors, or that broader metabolic benefits of physical activity in late midlife and older age are subtle and intertwined with other factors. Overall, the lack of robust associations does not rule out biological effects but underscores the complexity of isolating independent effects of LTPA and the need for rigorous statistical approaches in observational metabolomic research.

Associations between LTPA and metabolomic biomarkers appeared generally more evident in men than in women in both cross-sectional and longitudinal analyses. As the analyses were conducted separately in women and men to characterize within-sex associations, rather than to formally test whether these associations differed by sex, the stronger pattern of findings in men should be interpreted cautiously. The weaker findings in women may reflect biological differences in lipid and amino acid metabolism, body composition, or hormonal status, including menopause-related metabolic changes,^46^^,^^47^ although methodological factors may also have contributed, including sex differences in activity patterns, such as the type and intensity of LTPA, reporting accuracy, or limited power to detect modest associations after multiple-testing correction. Future studies specifically designed to examine sex differences are needed to clarify whether LTPA is differentially associated with metabolomic profiles and trajectories in women and men.

A major strength of this study is its 15-year longitudinal design with 4 clinical visits, enabling evaluation of temporal relationships between LTPA and the metabolome in a large, well-characterized birth cohort. Repeated metabolomic measurements using the Nightingale Health NMR metabolomics platform provided standardized, high-throughput quantification of 161 metabolomic biomarkers, ensuring reproducibility and clinical interpretability. LTPA was assessed twice using the validated KIHD questionnaire, improving the accuracy of self-reported activity and capturing changes over time. Adjustment for multiple confounders, consideration of nonlinear associations, and the usage of tenfold cross-validation further strengthened the analysis.

Our study has several limitations that should be considered. Generalizability may be limited by the cohort’s regional and temporal specificity. Loss to follow-up over 15 years, plausible in older cohorts because of worsening health and increasing mortality, may have introduced selection bias and reinforced healthy survivor bias, resulting in a healthier and potentially more active study sample. This was supported by baseline comparisons showing that completers were younger, had lower BMI, and had lower rates of diabetes and chronic disease burden than non-completers. In men, the lower proportion of low-LTPA participants among completers may have resulted in a healthier low-LTPA reference group, potentially attenuating rather than inflating the observed longitudinal differences. The high baseline LTPA levels may therefore reflect both the selected nature of the cohort and the characteristics of the LTPA assessment. LTPA was assessed using a self-reported 12-month recall questionnaire and is therefore subject to recall bias, social desirability, and potentially reduced accuracy in older adults because of age-related cognitive limitations.^48^ In addition, the KIHD questionnaire captures a broad range of leisure-time activities, not only structured exercise, which may have contributed to higher reported LTPA in some individuals. Although the KIHD questionnaire is based on the widely used Minnesota leisure-time activity questionnaire,^49^ its validity and reliability have not been specifically established in our age group to the same extent as in younger or middle-aged adults. Thus, it may be better suited to ranking, especially older participants, into broad activity categories than to precisely estimating absolute activity volume.^50^ Finally, the observational design precludes causal inference, and associations of LTPA with individual metabolomic biomarkers may be too subtle to detect under stringent multiple-testing correction, suggesting that larger sample sizes or more sensitive analytical methods may be necessary to capture these associations.

In conclusion, our study highlights a potential long-term association between LTPA and attenuated increases in triglyceride-enriched HDL module levels in men transitioning from late midlife to old age. This finding is consistent with a role for an active lifestyle in supporting metabolic health during aging in men. Future research should address the complex interplay of LTPA and other potential mediators and moderators of metabolic health.

## Supplementary Material

glag159_Supplementary_Data

## Data Availability

The data that support the findings of this study are available upon reasonable request.
