# Supplementary material for: Leisure-time physical activity and metabolomic trajectories over the transition from late midlife to old age
Source: J Gerontol A Biol Sci Med Sci. 2026 Jun 11;81(9):glag159. doi: 10.1093/gerona/glag159 (PMC13459948; doi:10.1093/gerona/glag159)
Supplement: Supplementary file 1 [file glag159_supplementary_data.docx]

**SUPPLEMENTAL MATERIAL**

**Leisure-time physical activity and metabolomic trajectories over the transition from late midlife to old age**

**FIGURES**

**eFigure 1.** The baseline association between leisure-time physical activity and albumin in women in the crude model (adjusted for age). p-value, the uncorrected significance level; q-value, the significance level after correcting for multiple testing. (Page 3)

**eFigure 2.** The baseline association between leisure-time physical activity (LTPA) and (a) triglyceride-enriched lipoproteins and fatty acids module (TG-FA module), (b), alanine (Ala), (c) phenylalanine (Phe), (d) glucose, (e) lactate, (f) pyruvate, (g) glycerol, (h) glycoprotein acetyls (GlycA), and (i) degree of fatty acid unsaturation (Unsaturation) in men in the crude model (adjusted for age). p-value, the uncorrected significance level; q-value, the significance level after correcting for multiple testing. (Page 4)

**TABLES**

**eTable 1.** Complete list of all 250 metabolomic biomarkers, with quality metrics and module assignments from the combined-sex Weighted Gene Co-expression Network Analysis. Module assignments are provided for the 161 biomarkers retained in the analysis. Of the original 250 biomarkers, 89 were excluded: 15 due to values below the limit of quantification (BLOQ) and 74 to avoid redundancy and to focus on absolute concentrations by excluding ratios and relative (%) lipoprotein measures. (Page 5-13)

**eTable 2.** The complete list of metabolomic biomarkers assigned to different modules based on Weighted Gene Co-Expression Network Analysis, and individual metabolomic biomarkers not assigned to any specific module. (Page 14-16)

**eTable 3.** Module membership of the 161 retained metabolomic biomarkers across the primary combined-sex Weighted Gene Co-expression Network Analysis network (WGCNA) (n=1890) and sex-stratified sensitivity networks in men (n=884) and women (n=1006). The women-only network identified 12 modules, whereas the combined-sex and men-only networks identified 11 modules, due to subdivision of the Large HDL particle module into two women-specific clusters (M5 and M6). (Page 17-22)

**eTable 4.** Baseline characteristics of women who attended the final visit (completers, n=413) versus those who did not (non-completers, n=553). (Page 23)

**eTable 5.** Baseline characteristics of men who attended the final visit (completers, n=327) versus those who did not (non-completers, n=523). (Page 24)

**eTable 6.** P-values for nominal associations between the volume of leisure-time physical activity and metabolomic modules and biomarkers in late midlife (baseline) in the crude and fully adjusted model in women and men. (Page 25)

**eTable 7.** Q-values (multiple-testing corrected p-values) for associations between the volume of leisure-time physical activity and metabolomic modules and biomarkers in late midlife (baseline) in the crude and fully adjusted model in women and men. (Page 26)

**eTable 8.** Age x Leisure-time physical activity interaction p-values for longitudinal metabolomic trajectories of modules and biomarkers in women and men before (p-value) and after (q-value) correcting for multiple testing. (Page 27)

**eTable 9.** Pairwise contrasts between low, moderate and high leisure-time physical activity (LTPA) groups in men for metabolomic biomarker modules with a nominally significant age × LTPA interaction (p<0.05), showing uncorrected p-values and multiple-testing-corrected q-values. (Page 28)

**eTable 10.** Age x Leisure-time physical activity change interaction p-values for longitudinal metabolomic trajectories of modules and biomarkers in women and men before (p-value) and after (q-value) correcting for multiple testing. (Page 29)

**eFigure 1. The baseline association between leisure-time physical activity and albumin in women in the crude model (adjusted for age). p-value, the uncorrected significance level; q-value, the significance level after correcting for multiple testing.**

**eFigure 2. The baseline association between leisure-time physical activity (LTPA) and (a) triglyceride-enriched lipoproteins and fatty acids module (TG-FA module), (b), alanine (Ala), (c) phenylalanine (Phe), (d) glucose, (e) lactate, (f) pyruvate, (g) glycerol, (h) glycoprotein acetyls (GlycA), and (i) degree of fatty acid unsaturation (Unsaturation) in men in the crude model (adjusted for age). p-value, the uncorrected significance level; q-value, the significance level after correcting for multiple testing.**

**eTable 1. Complete list of all 250 metabolomic biomarkers, with quality metrics and module assignments from the combined-sex Weighted Gene Co-expression Network Analysis. Module assignments are provided for the 161 biomarkers retained in the analysis. Of the original 250 biomarkers, 89 were excluded: 15 due to values below the limit of quantification (BLOQ) and 74 to avoid redundancy and to focus on absolute concentrations by excluding ratios and relative (%) lipoprotein measures.**

|  |  |  | **BLOQ values** | | **Outlier values excluded** | | | | **Exclusion reason** | |
| --- | --- | --- | --- | --- | --- | --- | --- | --- | --- | --- |
| **Biomarker** | **In analysis** | **Module** | **n** | **%** | | **n** | **%** |  | |  |
| Acetate | Yes | Unassigned | 2 | 0.04 | | 23 | 0.49 |  | |  |
| Acetoacetate | Yes | Unassigned | 541 | 11.51 | | 26 | 0.554 |  | |  |
| Acetone | Yes | Unassigned | 0 | 0.0 | | 21 | 0.447 |  | |  |
| Alanine | Yes | Unassigned | 0 | 0.0 | | 1 | 0.021 |  | |  |
| Albumin | Yes | Unassigned | 0 | 0.0 | | 4 | 0.085 |  | |  |
| Citrate | Yes | Unassigned | 0 | 0.0 | | 0 | 0.0 |  | |  |
| Creatinine | Yes | Unassigned | 6 | 0.13 | | 17 | 0.364 |  | |  |
| DHA | Yes | Unassigned | 1 | 0.02 | | 13 | 0.277 |  | |  |
| Glutamine | Yes | Unassigned | 0 | 0.0 | | 1 | 0.021 |  | |  |
| Glucose | Yes | Unassigned | 1 | 0.02 | | 30 | 0.639 |  | |  |
| Glycine | Yes | Unassigned | 0 | 0.0 | | 3 | 0.065 |  | |  |
| GlycA | Yes | Unassigned | 0 | 0.0 | | 4 | 0.085 |  | |  |
| Glycerol | Yes | Unassigned | 53 | 1.13 | | 5 | 0.118 |  | |  |
| Histidine | Yes | Unassigned | 0 | 0.0 | | 2 | 0.043 |  | |  |
| LDL size | Yes | Unassigned | 0 | 0.0 | | 4 | 0.085 |  | |  |
| Lactate | Yes | Unassigned | 1 | 0.02 | | 5 | 0.107 |  | |  |
| Omega-3 | Yes | Unassigned | 1 | 0.02 | | 8 | 0.17 |  | |  |
| Phenylalanine | Yes | Unassigned | 0 | 0.0 | | 6 | 0.128 |  | |  |
| Pyruvate | Yes | Unassigned | 0 | 0.0 | | 2 | 0.043 |  | |  |
| Tyrosine | Yes | Unassigned | 0 | 0.0 | | 2 | 0.043 |  | |  |
| Unsaturation | Yes | Unassigned | 1 | 0.02 | | 4 | 0.085 |  | |  |
| bOHbutyrate | Yes | Unassigned | 14 | 0.3 | | 26 | 0.568 |  | |  |
| ApoB | Yes | LDL-ApoB module | 0 | 0.0 | | 3 | 0.064 |  | |  |
| Clinical LDL-C | Yes | LDL-ApoB module | 0 | 0.0 | | 1 | 0.021 |  | |  |
| IDL-C | Yes | LDL-ApoB module | 0 | 0.0 | | 0 | 0.0 |  | |  |
| IDL-CE | Yes | LDL-ApoB module | 0 | 0.0 | | 0 | 0.0 |  | |  |
| IDL-FC | Yes | LDL-ApoB module | 0 | 0.0 | | 1 | 0.021 |  | |  |
| IDL-L | Yes | LDL-ApoB module | 0 | 0.0 | | 1 | 0.021 |  | |  |
| IDL-P | Yes | LDL-ApoB module | 0 | 0.0 | | 1 | 0.021 |  | |  |
| IDL-PL | Yes | LDL-ApoB module | 0 | 0.0 | | 1 | 0.021 |  | |  |
| L-LDL-C | Yes | LDL-ApoB module | 0 | 0.0 | | 1 | 0.021 |  | |  |
| L-LDL-CE | Yes | LDL-ApoB module | 0 | 0.0 | | 2 | 0.043 |  | |  |
| L-LDL-FC | Yes | LDL-ApoB module | 0 | 0.0 | | 1 | 0.021 |  | |  |
| L-LDL-L | Yes | LDL-ApoB module | 0 | 0.0 | | 1 | 0.021 |  | |  |
| L-LDL-P | Yes | LDL-ApoB module | 0 | 0.0 | | 2 | 0.043 |  | |  |
| L-LDL-PL | Yes | LDL-ApoB module | 0 | 0.0 | | 1 | 0.021 |  | |  |
| Linoleic acid | Yes | LDL-ApoB module | 1 | 0.02 | | 2 | 0.043 |  | |  |
| LDL-C | Yes | LDL-ApoB module | 0 | 0.0 | | 1 | 0.021 |  | |  |
| LDL-CE | Yes | LDL-ApoB module | 0 | 0.0 | | 1 | 0.021 |  | |  |
| LDL-FC | Yes | LDL-ApoB module | 0 | 0.0 | | 1 | 0.021 |  | |  |
| LDL-L | Yes | LDL-ApoB module | 0 | 0.0 | | 1 | 0.021 |  | |  |
| LDL-P | Yes | LDL-ApoB module | 0 | 0.0 | | 2 | 0.043 |  | |  |
| LDL-PL | Yes | LDL-ApoB module | 0 | 0.0 | | 1 | 0.021 |  | |  |
| M-LDL-C | Yes | LDL-ApoB module | 0 | 0.0 | | 2 | 0.043 |  | |  |
| M-LDL-CE | Yes | LDL-ApoB module | 0 | 0.0 | | 3 | 0.064 |  | |  |
| M-LDL-FC | Yes | LDL-ApoB module | 0 | 0.0 | | 1 | 0.021 |  | |  |
| M-LDL-L | Yes | LDL-ApoB module | 0 | 0.0 | | 1 | 0.021 |  | |  |
| M-LDL-P | Yes | LDL-ApoB module | 0 | 0.0 | | 3 | 0.064 |  | |  |
| M-LDL-PL | Yes | LDL-ApoB module | 0 | 0.0 | | 1 | 0.021 |  | |  |
| M-VLDL-C | Yes | LDL-ApoB module | 1 | 0.02 | | 2 | 0.043 |  | |  |
| M-VLDL-CE | Yes | LDL-ApoB module | 10 | 0.21 | | 1 | 0.021 |  | |  |
| Omega-6 | Yes | LDL-ApoB module | 1 | 0.02 | | 1 | 0.021 |  | |  |
| PUFA | Yes | LDL-ApoB module | 1 | 0.02 | | 2 | 0.043 |  | |  |
| Remnant-C | Yes | LDL-ApoB module | 0 | 0.0 | | 2 | 0.043 |  | |  |
| S-LDL-C | Yes | LDL-ApoB module | 0 | 0.0 | | 1 | 0.021 |  | |  |
| S-LDL-CE | Yes | LDL-ApoB module | 0 | 0.0 | | 3 | 0.064 |  | |  |
| S-LDL-FC | Yes | LDL-ApoB module | 1 | 0.02 | | 0 | 0.0 |  | |  |
| S-LDL-L | Yes | LDL-ApoB module | 0 | 0.0 | | 1 | 0.021 |  | |  |
| S-LDL-P | Yes | LDL-ApoB module | 0 | 0.0 | | 3 | 0.064 |  | |  |
| S-LDL-PL | Yes | LDL-ApoB module | 0 | 0.0 | | 1 | 0.021 |  | |  |
| Sphingomyelins | Yes | LDL-ApoB module | 1 | 0.02 | | 1 | 0.021 |  | |  |
| Total-C | Yes | LDL-ApoB module | 0 | 0.0 | | 1 | 0.021 |  | |  |
| Total-CE | Yes | LDL-ApoB module | 0 | 0.0 | | 0 | 0.0 |  | |  |
| Total-FC | Yes | LDL-ApoB module | 0 | 0.0 | | 1 | 0.021 |  | |  |
| Total-L | Yes | LDL-ApoB module | 0 | 0.0 | | 2 | 0.043 |  | |  |
| XS-VLDL-C | Yes | LDL-ApoB module | 0 | 0.0 | | 3 | 0.064 |  | |  |
| XS-VLDL-CE | Yes | LDL-ApoB module | 0 | 0.0 | | 2 | 0.043 |  | |  |
| non-HDL-C | Yes | LDL-ApoB module | 0 | 0.0 | | 1 | 0.021 |  | |  |
| L-VLDL-FC | Yes | Large VLDL module | 112 | 2.38 | | 13 | 0.277 |  | |  |
| L-VLDL-L | Yes | Large VLDL module | 2 | 0.04 | | 18 | 0.383 |  | |  |
| L-VLDL-P | Yes | Large VLDL module | 0 | 0.0 | | 16 | 0.341 |  | |  |
| L-VLDL-PL | Yes | Large VLDL module | 141 | 3.0 | | 14 | 0.298 |  | |  |
| L-VLDL-TG | Yes | Large VLDL module | 21 | 0.45 | | 19 | 0.405 |  | |  |
| M-VLDL-TG | Yes | Large VLDL module | 1 | 0.02 | | 13 | 0.277 |  | |  |
| Total-TG | Yes | Large VLDL module | 0 | 0.0 | | 16 | 0.341 |  | |  |
| VLDL size | Yes | Large VLDL module | 0 | 0.0 | | 3 | 0.064 |  | |  |
| VLDL-TG | Yes | Large VLDL module | 0 | 0.0 | | 19 | 0.405 |  | |  |
| XL-VLDL-L | Yes | Large VLDL module | 63 | 1.34 | | 18 | 0.383 |  | |  |
| XL-VLDL-P | Yes | Large VLDL module | 0 | 0.0 | | 19 | 0.405 |  | |  |
| XL-VLDL-PL | Yes | Large VLDL module | 610 | 12.98 | | 17 | 0.362 |  | |  |
| XL-VLDL-TG | Yes | Large VLDL module | 326 | 6.94 | | 19 | 0.405 |  | |  |
| XXL-VLDL-P | Yes | Large VLDL module | 0 | 0.0 | | 26 | 0.554 |  | |  |
| ApoA1 | Yes | HDL-ApoA1 module | 0 | 0.0 | | 1 | 0.021 |  | |  |
| HDL-C | Yes | HDL-ApoA1 module | 0 | 0.0 | | 0 | 0.0 |  | |  |
| HDL-CE | Yes | HDL-ApoA1 module | 0 | 0.0 | | 0 | 0.0 |  | |  |
| HDL-FC | Yes | HDL-ApoA1 module | 0 | 0.0 | | 1 | 0.021 |  | |  |
| HDL-L | Yes | HDL-ApoA1 module | 0 | 0.0 | | 1 | 0.021 |  | |  |
| HDL-P | Yes | HDL-ApoA1 module | 0 | 0.0 | | 3 | 0.064 |  | |  |
| HDL-PL | Yes | HDL-ApoA1 module | 0 | 0.0 | | 2 | 0.043 |  | |  |
| M-HDL-C | Yes | HDL-ApoA1 module | 0 | 0.0 | | 1 | 0.021 |  | |  |
| M-HDL-CE | Yes | HDL-ApoA1 module | 0 | 0.0 | | 1 | 0.021 |  | |  |
| M-HDL-FC | Yes | HDL-ApoA1 module | 0 | 0.0 | | 1 | 0.021 |  | |  |
| M-HDL-L | Yes | HDL-ApoA1 module | 0 | 0.0 | | 2 | 0.043 |  | |  |
| M-HDL-P | Yes | HDL-ApoA1 module | 0 | 0.0 | | 1 | 0.021 |  | |  |
| M-HDL-PL | Yes | HDL-ApoA1 module | 0 | 0.0 | | 2 | 0.043 |  | |  |
| Total-P | Yes | HDL-ApoA1 module | 0 | 0.0 | | 2 | 0.043 |  | |  |
| L-VLDL-C | Yes | Small VLDL module | 35 | 0.74 | | 9 | 0.192 |  | |  |
| L-VLDL-CE | Yes | Small VLDL module | 66 | 1.4 | | 6 | 0.128 |  | |  |
| M-VLDL-FC | Yes | Small VLDL module | 2 | 0.04 | | 3 | 0.064 |  | |  |
| M-VLDL-L | Yes | Small VLDL module | 0 | 0.0 | | 6 | 0.128 |  | |  |
| M-VLDL-P | Yes | Small VLDL module | 0 | 0.0 | | 4 | 0.085 |  | |  |
| M-VLDL-PL | Yes | Small VLDL module | 1 | 0.02 | | 4 | 0.085 |  | |  |
| S-VLDL-C | Yes | Small VLDL module | 0 | 0.0 | | 4 | 0.085 |  | |  |
| S-VLDL-CE | Yes | Small VLDL module | 0 | 0.0 | | 4 | 0.085 |  | |  |
| S-VLDL-FC | Yes | Small VLDL module | 0 | 0.0 | | 4 | 0.085 |  | |  |
| S-VLDL-L | Yes | Small VLDL module | 0 | 0.0 | | 4 | 0.085 |  | |  |
| S-VLDL-P | Yes | Small VLDL module | 0 | 0.0 | | 3 | 0.064 |  | |  |
| S-VLDL-PL | Yes | Small VLDL module | 0 | 0.0 | | 4 | 0.085 |  | |  |
| VLDL-C | Yes | Small VLDL module | 0 | 0.0 | | 5 | 0.106 |  | |  |
| VLDL-CE | Yes | Small VLDL module | 0 | 0.0 | | 2 | 0.043 |  | |  |
| VLDL-FC | Yes | Small VLDL module | 0 | 0.0 | | 10 | 0.213 |  | |  |
| VLDL-L | Yes | Small VLDL module | 0 | 0.0 | | 12 | 0.255 |  | |  |
| VLDL-P | Yes | Small VLDL module | 0 | 0.0 | | 5 | 0.106 |  | |  |
| VLDL-PL | Yes | Small VLDL module | 0 | 0.0 | | 9 | 0.192 |  | |  |
| XL-VLDL-C | Yes | Small VLDL module | 115 | 2.45 | | 12 | 0.255 |  | |  |
| XL-VLDL-CE | Yes | Small VLDL module | 180 | 3.83 | | 7 | 0.149 |  | |  |
| HDL size | Yes | Large HDL module | 0 | 0.0 | | 1 | 0.021 |  | |  |
| L-HDL-C | Yes | Large HDL module | 7 | 0.15 | | 0 | 0.0 |  | |  |
| L-HDL-CE | Yes | Large HDL module | 11 | 0.23 | | 0 | 0.0 |  | |  |
| L-HDL-FC | Yes | Large HDL module | 29 | 0.62 | | 0 | 0.0 |  | |  |
| L-HDL-L | Yes | Large HDL module | 0 | 0.0 | | 0 | 0.0 |  | |  |
| L-HDL-P | Yes | Large HDL module | 0 | 0.0 | | 0 | 0.0 |  | |  |
| L-HDL-PL | Yes | Large HDL module | 1 | 0.02 | | 1 | 0.021 |  | |  |
| XL-HDL-C | Yes | Large HDL module | 10 | 0.21 | | 0 | 0.0 |  | |  |
| XL-HDL-CE | Yes | Large HDL module | 16 | 0.34 | | 0 | 0.0 |  | |  |
| XL-HDL-FC | Yes | Large HDL module | 108 | 2.3 | | 5 | 0.106 |  | |  |
| XL-HDL-L | Yes | Large HDL module | 1 | 0.02 | | 1 | 0.021 |  | |  |
| XL-HDL-P | Yes | Large HDL module | 0 | 0.0 | | 1 | 0.021 |  | |  |
| XL-HDL-PL | Yes | Large HDL module | 145 | 3.09 | | 3 | 0.064 |  | |  |
| IDL-TG | Yes | TG-FA module | 0 | 0.0 | | 10 | 0.213 |  | |  |
| L-LDL-TG | Yes | TG-FA module | 0 | 0.0 | | 7 | 0.149 |  | |  |
| LDL-TG | Yes | TG-FA module | 0 | 0.0 | | 6 | 0.128 |  | |  |
| M-LDL-TG | Yes | TG-FA module | 0 | 0.0 | | 7 | 0.149 |  | |  |
| MUFA | Yes | TG-FA module | 1 | 0.02 | | 11 | 0.234 |  | |  |
| S-HDL-TG | Yes | TG-FA module | 9 | 0.19 | | 3 | 0.064 |  | |  |
| S-LDL-TG | Yes | TG-FA module | 124 | 2.64 | | 12 | 0.255 |  | |  |
| S-VLDL-TG | Yes | TG-FA module | 0 | 0.0 | | 9 | 0.192 |  | |  |
| SFA | Yes | TG-FA module | 1 | 0.02 | | 7 | 0.149 |  | |  |
| Total-FA | Yes | TG-FA module | 1 | 0.02 | | 4 | 0.085 |  | |  |
| XS-VLDL-TG | Yes | TG-FA module | 0 | 0.0 | | 5 | 0.106 |  | |  |
| Isoleucine | Yes | BCAA module | 0 | 0.0 | | 6 | 0.128 |  | |  |
| Leucine | Yes | BCAA module | 1 | 0.02 | | 4 | 0.085 |  | |  |
| Total BCAA | Yes | BCAA module | 1 | 0.02 | | 3 | 0.064 |  | |  |
| Valine | Yes | BCAA module | 0 | 0.0 | | 0 | 0.0 |  | |  |
| Cholines | Yes | Phospholipid module | 1 | 0.02 | | 0 | 0.0 |  | |  |
| Phosphatidylc | Yes | Phospholipid module | 1 | 0.02 | | 0 | 0.0 |  | |  |
| Phosphoglyc | Yes | Phospholipid module | 1 | 0.02 | | 1 | 0.021 |  | |  |
| Total-PL | Yes | Phospholipid module | 0 | 0.0 | | 0 | 0.0 |  | |  |
| XS-VLDL-FC | Yes | Extra-small VLDL module | 0 | 0.0 | | 4 | 0.085 |  | |  |
| XS-VLDL-L | Yes | Extra-small VLDL module | 0 | 0.0 | | 3 | 0.064 |  | |  |
| XS-VLDL-P | Yes | Extra-small VLDL module | 0 | 0.0 | | 4 | 0.085 |  | |  |
| XS-VLDL-PL | Yes | Extra-small VLDL module | 0 | 0.0 | | 3 | 0.064 |  | |  |
| S-HDL-C | Yes | Small HDL module | 0 | 0.0 | | 5 | 0.106 |  | |  |
| S-HDL-CE | Yes | Small HDL module | 0 | 0.0 | | 5 | 0.106 |  | |  |
| S-HDL-FC | Yes | Small HDL module | 0 | 0.0 | | 3 | 0.064 |  | |  |
| S-HDL-L | Yes | Small HDL module | 0 | 0.0 | | 3 | 0.064 |  | |  |
| S-HDL-P | Yes | Small HDL module | 0 | 0.0 | | 5 | 0.106 |  | |  |
| S-HDL-PL | Yes | Small HDL module | 0 | 0.0 | | 3 | 0.064 |  | |  |
| HDL-TG | Yes | HDL-TG module | 0 | 0.0 | | 6 | 0.128 |  | |  |
| L-HDL-TG | Yes | HDL-TG module | 18 | 0.38 | | 10 | 0.213 |  | |  |
| M-HDL-TG | Yes | HDL-TG module | 7 | 0.15 | | 3 | 0.064 |  | |  |
| XL-HDL-TG | No | -- | n/a | n/a | | n/a | n/a | >=20% BLOQ | |  |
| XL-HDL-TG % | No | -- | n/a | n/a | | n/a | n/a | >=20% BLOQ & ratio/percentage derivative | |  |
| XL-VLDL-FC | No | -- | n/a | n/a | | n/a | n/a | >=20% BLOQ | |  |
| XL-VLDL-FC % | No | -- | n/a | n/a | | n/a | n/a | >=20% BLOQ & ratio/percentage derivative | |  |
| XXL-VLDL-C | No | -- | n/a | n/a | | n/a | n/a | >=20% BLOQ | |  |
| XXL-VLDL-C % | No | -- | n/a | n/a | | n/a | n/a | >=20% BLOQ & ratio/percentage derivative | |  |
| XXL-VLDL-CE | No | -- | n/a | n/a | | n/a | n/a | >=20% BLOQ | |  |
| XXL-VLDL-CE % | No | -- | n/a | n/a | | n/a | n/a | >=20% BLOQ & ratio/percentage derivative | |  |
| XXL-VLDL-FC | No | -- | n/a | n/a | | n/a | n/a | >=20% BLOQ | |  |
| XXL-VLDL-FC % | No | -- | n/a | n/a | | n/a | n/a | >=20% BLOQ & ratio/percentage derivative | |  |
| XXL-VLDL-L | No | -- | n/a | n/a | | n/a | n/a | >=20% BLOQ | |  |
| XXL-VLDL-PL | No | -- | n/a | n/a | | n/a | n/a | >=20% BLOQ | |  |
| XXL-VLDL-PL % | No | -- | n/a | n/a | | n/a | n/a | >=20% BLOQ & ratio/percentage derivative | |  |
| XXL-VLDL-TG | No | -- | n/a | n/a | | n/a | n/a | >=20% BLOQ | |  |
| XXL-VLDL-TG % | No | -- | n/a | n/a | | n/a | n/a | >=20% BLOQ & ratio/percentage derivative | |  |
| ApoB/ApoA1 | No | -- | n/a | n/a | | n/a | n/a | ratio/percentage derivative | |  |
| DHA % | No | -- | n/a | n/a | | n/a | n/a | ratio/percentage derivative | |  |
| IDL-C % | No | -- | n/a | n/a | | n/a | n/a | ratio/percentage derivative | |  |
| IDL-CE % | No | -- | n/a | n/a | | n/a | n/a | ratio/percentage derivative | |  |
| IDL-FC % | No | -- | n/a | n/a | | n/a | n/a | ratio/percentage derivative | |  |
| IDL-PL % | No | -- | n/a | n/a | | n/a | n/a | ratio/percentage derivative | |  |
| IDL-TG % | No | -- | n/a | n/a | | n/a | n/a | ratio/percentage derivative | |  |
| L-HDL-C % | No | -- | n/a | n/a | | n/a | n/a | ratio/percentage derivative | |  |
| L-HDL-CE % | No | -- | n/a | n/a | | n/a | n/a | ratio/percentage derivative | |  |
| L-HDL-FC % | No | -- | n/a | n/a | | n/a | n/a | ratio/percentage derivative | |  |
| L-HDL-PL % | No | -- | n/a | n/a | | n/a | n/a | ratio/percentage derivative | |  |
| L-HDL-TG % | No | -- | n/a | n/a | | n/a | n/a | ratio/percentage derivative | |  |
| L-LDL-C % | No | -- | n/a | n/a | | n/a | n/a | ratio/percentage derivative | |  |
| L-LDL-CE % | No | -- | n/a | n/a | | n/a | n/a | ratio/percentage derivative | |  |
| L-LDL-FC % | No | -- | n/a | n/a | | n/a | n/a | ratio/percentage derivative | |  |
| L-LDL-PL % | No | -- | n/a | n/a | | n/a | n/a | ratio/percentage derivative | |  |
| L-LDL-TG % | No | -- | n/a | n/a | | n/a | n/a | ratio/percentage derivative | |  |
| L-VLDL-C % | No | -- | n/a | n/a | | n/a | n/a | ratio/percentage derivative | |  |
| L-VLDL-CE % | No | -- | n/a | n/a | | n/a | n/a | ratio/percentage derivative | |  |
| L-VLDL-FC % | No | -- | n/a | n/a | | n/a | n/a | ratio/percentage derivative | |  |
| L-VLDL-PL % | No | -- | n/a | n/a | | n/a | n/a | ratio/percentage derivative | |  |
| L-VLDL-TG % | No | -- | n/a | n/a | | n/a | n/a | ratio/percentage derivative | |  |
| Linoleic acid % | No | -- | n/a | n/a | | n/a | n/a | ratio/percentage derivative | |  |
| M-HDL-C % | No | -- | n/a | n/a | | n/a | n/a | ratio/percentage derivative | |  |
| M-HDL-CE % | No | -- | n/a | n/a | | n/a | n/a | ratio/percentage derivative | |  |
| M-HDL-FC % | No | -- | n/a | n/a | | n/a | n/a | ratio/percentage derivative | |  |
| M-HDL-PL % | No | -- | n/a | n/a | | n/a | n/a | ratio/percentage derivative | |  |
| M-HDL-TG % | No | -- | n/a | n/a | | n/a | n/a | ratio/percentage derivative | |  |
| M-LDL-C % | No | -- | n/a | n/a | | n/a | n/a | ratio/percentage derivative | |  |
| M-LDL-CE % | No | -- | n/a | n/a | | n/a | n/a | ratio/percentage derivative | |  |
| M-LDL-FC % | No | -- | n/a | n/a | | n/a | n/a | ratio/percentage derivative | |  |
| M-LDL-PL % | No | -- | n/a | n/a | | n/a | n/a | ratio/percentage derivative | |  |
| M-LDL-TG % | No | -- | n/a | n/a | | n/a | n/a | ratio/percentage derivative | |  |
| M-VLDL-C % | No | -- | n/a | n/a | | n/a | n/a | ratio/percentage derivative | |  |
| M-VLDL-CE % | No | -- | n/a | n/a | | n/a | n/a | ratio/percentage derivative | |  |
| M-VLDL-FC % | No | -- | n/a | n/a | | n/a | n/a | ratio/percentage derivative | |  |
| M-VLDL-PL % | No | -- | n/a | n/a | | n/a | n/a | ratio/percentage derivative | |  |
| M-VLDL-TG % | No | -- | n/a | n/a | | n/a | n/a | ratio/percentage derivative | |  |
| MUFA % | No | -- | n/a | n/a | | n/a | n/a | ratio/percentage derivative | |  |
| Omega-3 % | No | -- | n/a | n/a | | n/a | n/a | ratio/percentage derivative | |  |
| Omega-6 % | No | -- | n/a | n/a | | n/a | n/a | ratio/percentage derivative | |  |
| Omega-6/Omega-3 | No | -- | n/a | n/a | | n/a | n/a | ratio/percentage derivative | |  |
| PUFA % | No | -- | n/a | n/a | | n/a | n/a | ratio/percentage derivative | |  |
| PUFA/MUFA | No | -- | n/a | n/a | | n/a | n/a | ratio/percentage derivative | |  |
| S-HDL-C % | No | -- | n/a | n/a | | n/a | n/a | ratio/percentage derivative | |  |
| S-HDL-CE % | No | -- | n/a | n/a | | n/a | n/a | ratio/percentage derivative | |  |
| S-HDL-FC % | No | -- | n/a | n/a | | n/a | n/a | ratio/percentage derivative | |  |
| S-HDL-PL % | No | -- | n/a | n/a | | n/a | n/a | ratio/percentage derivative | |  |
| S-HDL-TG % | No | -- | n/a | n/a | | n/a | n/a | ratio/percentage derivative | |  |
| S-LDL-C % | No | -- | n/a | n/a | | n/a | n/a | ratio/percentage derivative | |  |
| S-LDL-CE % | No | -- | n/a | n/a | | n/a | n/a | ratio/percentage derivative | |  |
| S-LDL-FC % | No | -- | n/a | n/a | | n/a | n/a | ratio/percentage derivative | |  |
| S-LDL-PL % | No | -- | n/a | n/a | | n/a | n/a | ratio/percentage derivative | |  |
| S-LDL-TG % | No | -- | n/a | n/a | | n/a | n/a | ratio/percentage derivative | |  |
| S-VLDL-C % | No | -- | n/a | n/a | | n/a | n/a | ratio/percentage derivative | |  |
| S-VLDL-CE % | No | -- | n/a | n/a | | n/a | n/a | ratio/percentage derivative | |  |
| S-VLDL-FC % | No | -- | n/a | n/a | | n/a | n/a | ratio/percentage derivative | |  |
| S-VLDL-PL % | No | -- | n/a | n/a | | n/a | n/a | ratio/percentage derivative | |  |
| S-VLDL-TG % | No | -- | n/a | n/a | | n/a | n/a | ratio/percentage derivative | |  |
| SFA % | No | -- | n/a | n/a | | n/a | n/a | ratio/percentage derivative | |  |
| TG/PG | No | -- | n/a | n/a | | n/a | n/a | ratio/percentage derivative | |  |
| XL-HDL-C % | No | -- | n/a | n/a | | n/a | n/a | ratio/percentage derivative | |  |
| XL-HDL-CE % | No | -- | n/a | n/a | | n/a | n/a | ratio/percentage derivative | |  |
| XL-HDL-FC % | No | -- | n/a | n/a | | n/a | n/a | ratio/percentage derivative | |  |
| XL-HDL-PL % | No | -- | n/a | n/a | | n/a | n/a | ratio/percentage derivative | |  |
| XL-VLDL-C % | No | -- | n/a | n/a | | n/a | n/a | ratio/percentage derivative | |  |
| XL-VLDL-CE % | No | -- | n/a | n/a | | n/a | n/a | ratio/percentage derivative | |  |
| XL-VLDL-PL % | No | -- | n/a | n/a | | n/a | n/a | ratio/percentage derivative | |  |
| XL-VLDL-TG % | No | -- | n/a | n/a | | n/a | n/a | ratio/percentage derivative | |  |
| XS-VLDL-C % | No | -- | n/a | n/a | | n/a | n/a | ratio/percentage derivative | |  |
| XS-VLDL-CE % | No | -- | n/a | n/a | | n/a | n/a | ratio/percentage derivative | |  |
| XS-VLDL-FC % | No | -- | n/a | n/a | | n/a | n/a | ratio/percentage derivative | |  |
| XS-VLDL-PL % | No | -- | n/a | n/a | | n/a | n/a | ratio/percentage derivative | |  |
| XS-VLDL-TG % | No | -- | n/a | n/a | | n/a | n/a | ratio/percentage derivative | |  |

*Note. (1) LDL-ApoB module = Cholesterol-rich, ApoB containing LDL and IDL particle module, (2) Large VLDL module = Large triglyceride-rich VLDL particle module, (3) HDL-ApoA1 module = General and medium ApoA1 containing HDL particle module, (4) Small VLDL module = General and small VLDL particle module, (5) Large HDL module = Large HDL particle module, (6) TG-FA module = Triglyceride-enriched lipoproteins and fatty acids module, (7) BCAA module = Branched-chain amino acids module, (8) Phospholipid module = Phospholipid and choline module, (9) Extra-small VLDL module = Extra-small VLDL particle module, (10) Small HDL module = Small HDL particle module, and (11) HDL-TG module = triglyceride-enriched HDL module.*

*Abbreviations: ApoA1 = apolipoprotein A1; ApoB = apolipoprotein B; BCAA = branched-chain amino acid; bOHbutyrate = 3-hydroxybutyrate; C = cholesterol; CE = cholesteryl esters; DHA = docosahexaenoic acid; FA = fatty acid; FC = free cholesterol; ; GlycA = glycoprotein acetyls; HDL = high-density lipoprotein; IDL = intermediate-density lipoprotein; -L = total lipids; L- = large; LDL = low-density lipoprotein; M- =medium; MUFA = monounsaturated fatty acids; -P = concentration; -PL = phospholipids; Phosphatidylc = PC = Phosphatidylcholines; Phosphoglyc = PG = Phosphoglycerides; PL = phospholipids, PUFA = polyunsaturated fatty acids; S- = small; SFA = saturated fatty acids; TG = triglycerides; VLDL = very low-density lipoprotein; XL- = very large; XS- = extra-small; XXL- = extremely large.*

**eTable 2. The complete list of metabolomic biomarkers assigned to different modules based on Weighted Gene Co-Expression Network Analysis, and individual metabolomic biomarkers not assigned to any specific module.**

| **1) LDL-ApoB module** | **2) Large VLDL module** | **3) HDL-ApoA1 module** | **4) Small VLDL module** | **5) Large HDL module** | **6) TG-FA module** | **7) BCAA module** | **8) Phospholipid module** | **9) Extra-small VLDL module** | **10) Small HDL module** | **11) HDL-TG module** | **Individual metabolomic biomarkers** |  |
| --- | --- | --- | --- | --- | --- | --- | --- | --- | --- | --- | --- | --- |
| Total-C | Total-TG | HDL-C | VLDL-C | HDL size | LDL-TG | Total BCAA | Total-PL | XS-VLDL-P | S-HDL-P | HDL-TG | LDL size |  |
| Non-HDL-C | VLDL-TG | HDL-PL | VLDL-PL | XL-HDL-P | Total-FA | Isoleucine | Phosphoglycerides | XS-VLDL-L | S-HDL-L | L-HDL-TG | FA unsaturation |  |
| Remnant-C | VLDL size | HDL-CE | VLDL-CE | XL-HDL-L | MUFA | Leucine | Cholines | XS-VLDL-PL | S-HDL-PL | M-HDL-TG | Omega-3 |  |
| Clinical-LDL-C | XXL-VLDL-P | HDL-FC | VLDL-FC | XL-HDL-PL | SFA | Valine | Phosphatidylcholines | XS-VLDL-FC | S-HDL-C |  | Docosahexaenoic acid |  |
| LDL-C | XL-VLDL-P | HDL-L | VLDL-L | XL-HDL-C | S-VLDL-TG |  |  |  | S-HDL-CE |  | Alanine |  |
| LDL-PL | XL-VLDL-L | Total-P | VLDL-P | XL-HDL-CE | XS-VLDL-TG |  |  |  | S-HDL-FC |  | Glutamine |  |
| Total-CE | XL-VLDL-PL | HDL-P | XL-VLDL-C | XL-HDL-FC | IDL-TG |  |  |  |  |  | Glycine |  |
| LDL-CE | XL-VLDL-TG | ApoA1 | XL-VLDL-CE | L-HDL-P | L-LDL-TG |  |  |  |  |  | Histidine |  |
| Total-FC | L-VLDL-P | M-HDL-P | L-VLDL-C | L-HDL-L | M-LDL-TG |  |  |  |  |  | Phenylalanine |  |
| LDL-FC | L-VLDL-L | M-HDL-L | L-VLDL-CE | L-HDL-PL | S-LDL-TG |  |  |  |  |  | Tyrosine |  |
| Total-L | L-VLDL-PL | M-HDL-PL | M-VLDL-P | L-HDL-C | S-HDL-TG |  |  |  |  |  | Glucose |  |
| LDL-L | L-VLDL-FC | M-HDL-C | M-VLDL-L | L-HDL-CE |  |  |  |  |  |  | Lactate |  |
| LDL-P | L-VLDL-TG | M-HDL-CE | M-VLDL-PL | L-HDL-FC |  |  |  |  |  |  | Pyruvate |  |
| Sphingomyelins | M-VLDL-TG | M-HDL-FC | M-VLDL-FC |  |  |  |  |  |  |  | Citrate |  |
| ApoB |  |  | S-VLDL-P |  |  |  |  |  |  |  | Glycerol |  |
| Omega-6 |  |  | S-VLDL-L |  |  |  |  |  |  |  | 3-hydroxybutyrate | |
| PUFA |  |  | S-VLDL-PL |  |  |  |  |  |  |  | Acetate |  |
| Linoleic acid |  |  | S-VLDL-C |  |  |  |  |  |  |  | Acetoacetate |  |
| M-VLDL-C |  |  | S-VLDL-CE |  |  |  |  |  |  |  | Acetone |  |
| M-VLDL-CE |  |  | S-VLDL-FC |  |  |  |  |  |  |  | Creatinine |  |
| XS-VLDL-C |  |  |  |  |  |  |  |  |  |  | Albumin |  |
| XS-VLDL-CE |  |  |  |  |  |  |  |  |  |  | GlycA |  |
| IDL-P |  |  |  |  |  |  |  |  |  |  |  |  |
| IDL-L |  |  |  |  |  |  |  |  |  |  |  |  |
| IDL-PL |  |  |  |  |  |  |  |  |  |  |  |  |
| IDL-C |  |  |  |  |  |  |  |  |  |  |  |  |
| IDL-CE |  |  |  |  |  |  |  |  |  |  |  |  |
| IDL-FC |  |  |  |  |  |  |  |  |  |  |  |  |
| L-LDL-P |  |  |  |  |  |  |  |  |  |  |  |  |
| L-LDL-L |  |  |  |  |  |  |  |  |  |  |  |  |
| L-LDL-PL |  |  |  |  |  |  |  |  |  |  |  |  |
| L-LDL-C |  |  |  |  |  |  |  |  |  |  |  |  |
| L-LDL-CE |  |  |  |  |  |  |  |  |  |  |  |  |
| L-LDL-FC |  |  |  |  |  |  |  |  |  |  |  |  |
| M-LDL-P |  |  |  |  |  |  |  |  |  |  |  |  |
| M-LDL-L |  |  |  |  |  |  |  |  |  |  |  |  |
| M-LDL-PL |  |  |  |  |  |  |  |  |  |  |  |  |
| M-LDL-C |  |  |  |  |  |  |  |  |  |  |  |  |
| M-LDL-CE |  |  |  |  |  |  |  |  |  |  |  |  |
| M-LDL-FC |  |  |  |  |  |  |  |  |  |  |  |  |
| S-LDL-P |  |  |  |  |  |  |  |  |  |  |  |  |
| S-LDL-L |  |  |  |  |  |  |  |  |  |  |  |  |
| S-LDL-PL |  |  |  |  |  |  |  |  |  |  |  |  |
| S-LDL-C |  |  |  |  |  |  |  |  |  |  |  |  |
| S-LDL-CE |  |  |  |  |  |  |  |  |  |  |  |  |
| S-LDL-FC |  |  |  |  |  |  |  |  |  |  |  |  |

*Note. (1) LDL-ApoB module = Cholesterol-rich, ApoB containing LDL and IDL particle module, (2) Large VLDL module = Large triglyceride-rich VLDL particle module, (3) HDL-ApoA1 module = General and medium ApoA1 containing HDL particle module, (4) Small VLDL module = General and small VLDL particle module, (5) Large HDL module = Large HDL particle module, (6) TG-FA module = Triglyceride-enriched lipoproteins and fatty acids module, (7) BCAA module = Branched-chain amino acids module, (8) Phospholipid module = Phospholipid and choline module, (9) Extra-small VLDL module = Extra-small VLDL particle module, (10) Small HDL module = Small HDL particle module, and (11) HDL-TG module = triglyceride-enriched HDL module.*

*Abbreviations: ApoA1 = apolipoprotein A1; ApoB = apolipoprotein B; BCAA = branched-chain amino acid; C = cholesterol; CE = cholesteryl esters; FA = fatty acid; FC = free cholesterol; ; GlycA = glycoprotein acetyls; HDL = high-density lipoprotein; IDL = intermediate-density lipoprotein; -L = total lipids; L- = large; LDL = low-density lipoprotein; M- =medium; MUFA = monounsaturated fatty acids; -P = concentration; PL = phospholipids, PUFA = polyunsaturated fatty acids; S- = small; SFA = saturated fatty acids; TG = triglycerides; VLDL = very low-density lipoprotein; XL- = very large; XS- = extra-small; XXL- = extremely large.*

**eTable 3. Module membership of the 161 retained metabolomic biomarkers across the primary combined-sex Weighted Gene Co-expression Network Analysis network (WGCNA) (n=1890) and sex-stratified sensitivity networks in men (n=884) and women (n=1006). The women-only network identified 12 modules, whereas the combined-sex and men-only networks identified 11 modules, due to subdivision of the Large HDL particle module into two women-specific clusters (M5 and M6).**

| **Metabolomic biomarker** | **Combined network** | **Men-only network** | **Women-only network** |
| --- | --- | --- | --- |
| Acetate | Unassigned | Unassigned | Unassigned |
| Acetoacetate | Unassigned | Unassigned | Unassigned |
| Acetone | Unassigned | Unassigned | Unassigned |
| Alanine | Unassigned | Unassigned | Unassigned |
| Albumin | Unassigned | Unassigned | Unassigned |
| Citrate | Unassigned | Unassigned | Unassigned |
| Creatinine | Unassigned | Unassigned | Unassigned |
| DHA | Unassigned | Unassigned | Unassigned |
| Glutamine | Unassigned | Unassigned | Unassigned |
| Glucose | Unassigned | Unassigned | Unassigned |
| Glycine | Unassigned | Unassigned | Unassigned |
| GlycA | Unassigned | Unassigned | Unassigned |
| Glycerol | Unassigned | Unassigned | Unassigned |
| Histidine | Unassigned | Unassigned | Unassigned |
| LDL-size | Unassigned | Unassigned | Unassigned |
| Lactate | Unassigned | Unassigned | Unassigned |
| Omega_3 | Unassigned | Unassigned | Unassigned |
| Phenylalanine | Unassigned | Unassigned | Unassigned |
| Pyruvate | Unassigned | Unassigned | Unassigned |
| Tyrosine | Unassigned | Unassigned | Unassigned |
| Unsaturation | Unassigned | Unassigned | Unassigned |
| bOHbutyrate | Unassigned | Unassigned | Unassigned |
| ApoB | LDL-ApoB module | LDL-ApoB module (M1) | LDL-ApoB module (M2) |
| Clinical-LDL-C | LDL-ApoB module | LDL-ApoB module (M1) | LDL-ApoB module (M2) |
| IDL-C | LDL-ApoB module | LDL-ApoB module (M1) | LDL-ApoB module (M2) |
| IDL-CE | LDL-ApoB module | LDL-ApoB module (M1) | LDL-ApoB module (M2) |
| IDL-FC | LDL-ApoB module | LDL-ApoB module (M1) | LDL-ApoB module (M2) |
| IDL-L | LDL-ApoB module | LDL-ApoB module (M1) | LDL-ApoB module (M2) |
| IDL-P | LDL-ApoB module | LDL-ApoB module (M1) | LDL-ApoB module* (M10) |
| IDL-PL | LDL-ApoB module | Extra-small VLDL module* (M6) | LDL-ApoB module (M2) |
| LA | LDL-ApoB module | LDL-ApoB module (M1) | Phospholipid module* (M7) |
| LDL-C | LDL-ApoB module | LDL-ApoB module (M1) | LDL-ApoB module (M2) |
| LDL-CE | LDL-ApoB module | LDL-ApoB module (M1) | LDL-ApoB module (M2) |
| LDL-FC | LDL-ApoB module | LDL-ApoB module (M1) | LDL-ApoB module (M2) |
| LDL-L | LDL-ApoB module | LDL-ApoB module (M1) | LDL-ApoB module (M2) |
| LDL-P | LDL-ApoB module | LDL-ApoB module (M1) | LDL-ApoB module (M2) |
| LDL-PL | LDL-ApoB module | LDL-ApoB module (M1) | LDL-ApoB module (M2) |
| L-LDL-C | LDL-ApoB module | LDL-ApoB module (M1) | LDL-ApoB module (M2) |
| L-LDL-CE | LDL-ApoB module | LDL-ApoB module (M1) | LDL-ApoB module (M2) |
| L-LDL-FC | LDL-ApoB module | LDL-ApoB module (M1) | LDL-ApoB module (M2) |
| L-LDL-L | LDL-ApoB module | LDL-ApoB module (M1) | LDL-ApoB module (M2) |
| L-LDL-P | LDL-ApoB module | LDL-ApoB module (M1) | LDL-ApoB module (M2) |
| L-LDL-PL | LDL-ApoB module | LDL-ApoB module (M1) | LDL-ApoB module (M2) |
| M-LDL-C | LDL-ApoB module | LDL-ApoB module (M1) | LDL-ApoB module (M2) |
| M-LDL-CE | LDL-ApoB module | LDL-ApoB module (M1) | LDL-ApoB module (M2) |
| M-LDL-FC | LDL-ApoB module | LDL-ApoB module (M1) | LDL-ApoB module (M2) |
| M-LDL-L | LDL-ApoB module | LDL-ApoB module (M1) | LDL-ApoB module (M2) |
| M-LDL-P | LDL-ApoB module | LDL-ApoB module (M1) | LDL-ApoB module (M2) |
| M-LDL-PL | LDL-ApoB module | LDL-ApoB module (M1) | LDL-ApoB module (M2) |
| M-VLDL-C | LDL-ApoB module | LDL-ApoB module (M1) | LDL-ApoB module (M2) |
| M-VLDL-CE | LDL-ApoB module | LDL-ApoB module (M1) | LDL-ApoB module (M2) |
| Omega_6 | LDL-ApoB module | LDL-ApoB module (M1) | Phospholipid module* (M7) |
| PUFA | LDL-ApoB module | LDL-ApoB module (M1) | Phospholipid module* (M7) |
| Remnant_C | LDL-ApoB module | LDL-ApoB module (M1) | LDL-ApoB module* (M10) |
| S-LDL-C | LDL-ApoB module | LDL-ApoB module (M1) | LDL-ApoB module (M2) |
| S-LDL-CE | LDL-ApoB module | LDL-ApoB module (M1) | LDL-ApoB module (M2) |
| S-LDL-FC | LDL-ApoB module | LDL-ApoB module (M1) | LDL-ApoB module (M2) |
| S-LDL-L | LDL-ApoB module | LDL-ApoB module (M1) | LDL-ApoB module (M2) |
| S-LDL-P | LDL-ApoB module | LDL-ApoB module (M1) | LDL-ApoB module (M2) |
| S-LDL-PL | LDL-ApoB module | LDL-ApoB module (M1) | LDL-ApoB module (M2) |
| Sphingomyelins | LDL-ApoB module | LDL-ApoB module (M1) | LDL-ApoB module (M2) |
| Total_C | LDL-ApoB module | LDL-ApoB module (M1) | LDL-ApoB module (M2) |
| Total_CE | LDL-ApoB module | LDL-ApoB module (M1) | LDL-ApoB module (M2) |
| Total_FC | LDL-ApoB module | LDL-ApoB module (M1) | LDL-ApoB module (M2) |
| Total_L | LDL-ApoB module | LDL-ApoB module (M1) | Phospholipid module* (M7) |
| XS-VLDL-C | LDL-ApoB module | Extra-small VLDL module* (M6) | LDL-ApoB module* (M10) |
| XS-VLDL-CE | LDL-ApoB module | Extra-small VLDL module* (M6) | LDL-ApoB module* (M10) |
| non-HDL-C | LDL-ApoB module | LDL-ApoB module (M1) | LDL-ApoB module (M2) |
| L-VLDL-FC | Large VLDL module | Large VLDL module (M2) | Large VLDL module* (M1) |
| L-VLDL-L | Large VLDL module | Large VLDL module (M2) | Large VLDL module* (M1) |
| L-VLDL-P | Large VLDL module | Large VLDL module (M2) | Large VLDL module* (M1) |
| L-VLDL-PL | Large VLDL module | Large VLDL module (M2) | Large VLDL module* (M1) |
| L-VLDL-TG | Large VLDL module | Large VLDL module (M2) | Large VLDL module* (M1) |
| M-VLDL-TG | Large VLDL module | Large VLDL module (M2) | Large VLDL module* (M1) |
| Total_TG | Large VLDL module | Large VLDL module (M2) | Large VLDL module* (M1) |
| VLDL-TG | Large VLDL module | Large VLDL module (M2) | Large VLDL module* (M1) |
| VLDL-size | Large VLDL module | Large VLDL module (M2) | Large VLDL module* (M1) |
| XL-VLDL-L | Large VLDL module | Large VLDL module (M2) | Large VLDL module* (M1) |
| XL-VLDL-P | Large VLDL module | Large VLDL module (M2) | Large VLDL module* (M1) |
| XL-VLDL-PL | Large VLDL module | Large VLDL module (M2) | Large VLDL module* (M1) |
| XL-VLDL-TG | Large VLDL module | Large VLDL module (M2) | Large VLDL module* (M1) |
| XXL-VLDL-P | Large VLDL module | Large VLDL module (M2) | Large VLDL module* (M1) |
| ApoA1 | HDL-ApoA1 module | HDL-ApoA1 module (M4) | HDL-ApoA1 module (M3) |
| HDL-C | HDL-ApoA1 module | HDL-ApoA1 module (M4) | Large HDL module* (M5) |
| HDL-CE | HDL-ApoA1 module | HDL-ApoA1 module (M4) | Large HDL module* (M5) |
| HDL-FC | HDL-ApoA1 module | HDL-ApoA1 module (M4) | Large HDL module* (M5) |
| HDL-L | HDL-ApoA1 module | HDL-ApoA1 module (M4) | HDL-ApoA1 module (M3) |
| HDL-P | HDL-ApoA1 module | HDL-ApoA1 module (M4) | HDL-ApoA1 module (M3) |
| HDL-PL | HDL-ApoA1 module | HDL-ApoA1 module (M4) | HDL-ApoA1 module (M3) |
| M-HDL-C | HDL-ApoA1 module | HDL-ApoA1 module (M4) | HDL-ApoA1 module (M3) |
| M-HDL-CE | HDL-ApoA1 module | HDL-ApoA1 module (M4) | HDL-ApoA1 module (M3) |
| M-HDL-FC | HDL-ApoA1 module | HDL-ApoA1 module (M4) | HDL-ApoA1 module (M3) |
| M-HDL-L | HDL-ApoA1 module | HDL-ApoA1 module (M4) | HDL-ApoA1 module (M3) |
| M-HDL-P | HDL-ApoA1 module | HDL-ApoA1 module (M4) | HDL-ApoA1 module (M3) |
| M-HDL-PL | HDL-ApoA1 module | HDL-ApoA1 module (M4) | HDL-ApoA1 module (M3) |
| Total_P | HDL-ApoA1 module | Phospholipid module* (M7) | HDL-ApoA1 module (M3) |
| L-VLDL-C | Small VLDL module | Small VLDL module (M3) | Large VLDL module* (M1) |
| L-VLDL-CE | Small VLDL module | Small VLDL module (M3) | Small VLDL module (M4) |
| M-VLDL-FC | Small VLDL module | Small VLDL module (M3) | Small VLDL module (M4) |
| M-VLDL-L | Small VLDL module | Small VLDL module (M3) | Small VLDL module (M4) |
| M-VLDL-P | Small VLDL module | Small VLDL module (M3) | Small VLDL module (M4) |
| M-VLDL-PL | Small VLDL module | Small VLDL module (M3) | Small VLDL module (M4) |
| S-VLDL-C | Small VLDL module | Small VLDL module (M3) | Small VLDL module (M4) |
| S-VLDL-CE | Small VLDL module | Small VLDL module (M3) | Small VLDL module (M4) |
| S-VLDL-FC | Small VLDL module | Small VLDL module (M3) | Small VLDL module (M4) |
| S-VLDL-L | Small VLDL module | Small VLDL module (M3) | Small VLDL module (M4) |
| S-VLDL-P | Small VLDL module | Small VLDL module (M3) | Small VLDL module (M4) |
| S-VLDL-PL | Small VLDL module | Small VLDL module (M3) | Small VLDL module (M4) |
| VLDL-C | Small VLDL module | Small VLDL module (M3) | Small VLDL module (M4) |
| VLDL-CE | Small VLDL module | Small VLDL module (M3) | Small VLDL module (M4) |
| VLDL-FC | Small VLDL module | Small VLDL module (M3) | Small VLDL module (M4) |
| VLDL-L | Small VLDL module | Small VLDL module (M3) | Large VLDL module* (M1) |
| VLDL-P | Small VLDL module | Small VLDL module (M3) | Small VLDL module (M4) |
| VLDL-PL | Small VLDL module | Small VLDL module (M3) | Small VLDL module (M4) |
| XL-VLDL-C | Small VLDL module | Small VLDL module (M3) | Large VLDL module* (M1) |
| XL-VLDL-CE | Small VLDL module | Small VLDL module (M3) | Small VLDL module (M4) |
| HDL-size | Large HDL module | Large HDL module (M5) | Large HDL module* (M5) |
| L-HDL-C | Large HDL module | Large HDL module (M5) | Large HDL module* (M5) |
| L-HDL-CE | Large HDL module | Large HDL module (M5) | Large HDL module* (M5) |
| L-HDL-FC | Large HDL module | Large HDL module (M5) | Large HDL module* (M5) |
| L-HDL-L | Large HDL module | Large HDL module (M5) | Large HDL module* (M5) |
| L-HDL-P | Large HDL module | Large HDL module (M5) | Large HDL module* (M5) |
| L-HDL-PL | Large HDL module | Large HDL module (M5) | Large HDL module* (M5) |
| XL-HDL-C | Large HDL module | Large HDL module (M5) | Large HDL module (M6) |
| XL-HDL-CE | Large HDL module | Large HDL module (M5) | Large HDL module (M6) |
| XL-HDL-FC | Large HDL module | Large HDL module (M5) | Large HDL module (M6) |
| XL-HDL-L | Large HDL module | Large HDL module (M5) | Large HDL module (M6) |
| XL-HDL-P | Large HDL module | Large HDL module (M5) | Large HDL module (M6) |
| XL-HDL-PL | Large HDL module | Large HDL module (M5) | Large HDL module (M6) |
| IDL-TG | TG-FA module | TG-FA module (M9) | TG-FA module (M8) |
| LDL-TG | TG-FA module | TG-FA module (M9) | TG-FA module (M8) |
| L-LDL-TG | TG-FA module | TG-FA module (M9) | TG-FA module (M8) |
| MUFA | TG-FA module | TG-FA module (M9) | Large VLDL module* (M1) |
| M-LDL-TG | TG-FA module | TG-FA module (M9) | TG-FA module (M8) |
| SFA | TG-FA module | Small VLDL module (M3) | Phospholipid module* (M7) |
| S-HDL-TG | TG-FA module | TG-FA module (M9) | Large VLDL module* (M1) |
| S-LDL-TG | TG-FA module | TG-FA module (M9) | Large VLDL module* (M1) |
| S-VLDL-TG | TG-FA module | TG-FA module (M9) | Large VLDL module* (M1) |
| Total_FA | TG-FA module | Small VLDL module (M3) | Phospholipid module* (M7) |
| XS-VLDL-TG | TG-FA module | TG-FA module (M9) | TG-FA module (M8) |
| Isoleucine | BCAA module | BCAA module (M10) | BCAA module (M9) |
| Leucine | BCAA module | BCAA module (M10) | BCAA module (M9) |
| Total_BCAA | BCAA module | BCAA module (M10) | BCAA module (M9) |
| Valine | BCAA module | BCAA module (M10) | BCAA module (M9) |
| Cholines | Phospholipid module | Phospholipid module* (M7) | Phospholipid module* (M7) |
| Phosphatidylc | Phospholipid module | Phospholipid module* (M7) | Phospholipid module* (M7) |
| Phosphoglyc | Phospholipid module | Phospholipid module* (M7) | Phospholipid module* (M7) |
| Total_PL | Phospholipid module | Phospholipid module* (M7) | Phospholipid module* (M7) |
| XS-VLDL-FC | Extra-small VLDL module | Extra-small VLDL module* (M6) | LDL-ApoB module* (M10) |
| XS-VLDL-L | Extra-small VLDL module | Extra-small VLDL module* (M6) | LDL-ApoB module* (M10) |
| XS-VLDL-P | Extra-small VLDL module | Extra-small VLDL module* (M6) | LDL-ApoB module* (M10) |
| XS-VLDL-PL | Extra-small VLDL module | Extra-small VLDL module* (M6) | LDL-ApoB module* (M10) |
| S-HDL-C | Small HDL module | Small HDL module (M8) | Small HDL module (M11) |
| S-HDL-CE | Small HDL module | Small HDL module (M8) | Small HDL module (M11) |
| S-HDL-FC | Small HDL module | Phospholipid module* (M7) | Small HDL module (M11) |
| S-HDL-L | Small HDL module | Small HDL module (M8) | Small HDL module (M11) |
| S-HDL-P | Small HDL module | Small HDL module (M8) | Small HDL module (M11) |
| S-HDL-PL | Small HDL module | Small HDL module (M8) | Small HDL module (M11) |
| HDL-TG | HDL-TG module | HDL-TG module (M11) | HDL-TG module (M12) |
| L-HDL-TG | HDL-TG module | HDL-TG module (M11) | HDL-TG module (M12) |
| M-HDL-TG | HDL-TG module | HDL-TG module (M11) | HDL-TG module (M12) |

*Note. Module labels for the men-only and women-only networks reflect the biological content of each module, determined by mapping sex-stratified module numbers to the most corresponding combined-network module based on majority metabolite membership. Module numbers in parentheses (e.g., M12) are assigned by the WGCNA algorithm based on cluster size rank within each network and do not correspond across networks. An asterisk (*) indicates a biologically mixed module in which fewer than 75% of metabolites map to a single combined-network module.*

*Note. (1) LDL-ApoB module = Cholesterol-rich, ApoB containing LDL and IDL particle module, (2) Large VLDL module = Large triglyceride-rich VLDL particle module, (3) HDL-ApoA1 module = General and medium ApoA1 containing HDL particle module, (4) Small VLDL module = General and small VLDL particle module, (5) Large HDL module = Large HDL particle module, (6) TG-FA module = Triglyceride-enriched lipoproteins and fatty acids module, (7) BCAA module = Branched-chain amino acids module, (8) Phospholipid module = Phospholipid and choline module, (9) Extra-small VLDL module = Extra-small VLDL particle module, (10) Small HDL module = Small HDL particle module, and (11) HDL-TG module = triglyceride-enriched HDL module.*

*Abbreviations: ApoA1 = apolipoprotein A1; ApoB = apolipoprotein B; BCAA = branched-chain amino acid; C = cholesterol; CE = cholesteryl esters; FA = fatty acid; FC = free cholesterol; ; GlycA = glycoprotein acetyls; HDL = high-density lipoprotein; IDL = intermediate-density lipoprotein; -L = total lipids; L- = large; LDL = low-density lipoprotein; M- =medium; MUFA = monounsaturated fatty acids; -P = concentration; PL = phospholipids, PUFA = polyunsaturated fatty acids; S- = small; SFA = saturated fatty acids; TG = triglycerides; VLDL = very low-density lipoprotein; XL- = very large; XS- = extra-small; XXL- = extremely large.*

**eTable 4. Baseline characteristics of women who attended the final visit (completers, n=413) versus those who did not (non-completers, n=553).**

| **Variable** | **Completers** | **Non-completers** | **p-value** |
| --- | --- | --- | --- |
| Age, years, mean (SD) | 61.2 (2.9) | 61.9 (3.1) | <0.001 |
| Body mass index, kg/m², mean (SD) | 27.1 (4.5) | 28.1 (5.3) | 0.002 |
| Educational status, n (%) |  |  | 0.012 |
| Primary or less | 135 (32.7) | 236 (42.7) | — |
| Upper secondary | 72 (17.4) | 89 (16.1) | — |
| Lower tertiary | 112 (27.1) | 133 (24.1) | — |
| Upper tertiary | 94 (22.8) | 95 (17.2) | — |
| Smoking status, n (%) |  |  | <0.001 |
| Never | 248 (60.0) | 285 (51.5) | — |
| Former | 104 (25.2) | 132 (23.9) | — |
| Light | 28 (6.8) | 27 (4.9) | — |
| Regular | 33 (8.0) | 109 (19.7) | — |
| Alcohol use, n (%) |  |  | <0.001 |
| None | 12 (2.9) | 55 (9.9) | — |
| Light | 219 (53.0) | 303 (54.8) | — |
| Regular | 182 (44.1) | 195 (35.3) | — |
| Chronic diseases, n (%) |  |  | <0.001 |
| None | 232 (56.2) | 230 (41.6 | — |
| 1 disease | 140 (33.9) | 207 (37.4) | — |
| ≥2 diseases | 41 (9.9) | 116 (21.0) | — |
| Diabetes at baseline, yes n (%) | 35 (8.5) | 94 (17.0) | <0.001 |
| LTPA, METh/wk, mean (SD) | 43.2 (34.4) | 48.8 (42.7) | 0.026 |
| LTPA category, n (%) |  |  | 0.055 |
| Low (<16.7 METh/wk) | 77 (18.6) | 118 (21.3) | — |
| Moderate (16.7-50 METh/wk) | 210 (50.8) | 238 (43.0) | — |
| High (≥50 METh/wk) | 126 (30.5) | 197 (35.6) | — |
| Dietary intake, mean (SD) |  |  | — |
| Total energy, kJ/day | 8428.4 (2796.5) | 8567.0 (3015.3) | 0.461 |
| Protein, E% | 17.3 (2.4) | 17.4 (2.6) | 0.455 |
| Carbohydrates, E% | 47.6 (6.2) | 47.9 (7.0) | 0.434 |
| Fat, E% | 33.2 (4.9) | 33.1 (5.7) | 0.825 |
| Saturated fat, E% | 12.1 (2.4) | 12.1 (2.7) | 0.983 |
| PUFA, E% | 5.3 (1.3) | 5.3 (1.3) | 0.994 |
| MUFA, E% | 10.9 (2.0) | 10.9 (2.2) | 0.832 |
| Alcohol, E% | 2.1 (2.7) | 1.8 (2.7) | 0.079 |

*Note. p-values derived from independent samples t-tests for continuous variables and chi-square tests for categorical variables.*

*Abbreviations. SD = standard deviation; LTPA = leisure-time physical activity; PUFA = polyunsaturated fatty acids; MUFA = monounsaturated fatty acids.*

**eTable 5. Baseline characteristics of men who attended the final visit (completers, n=327) versus those who did not (non-completers, n=523).**

| **Variable** | **Completers** | **Non-completers** | **p-value** |
| --- | --- | --- | --- |
| Age, years, mean (SD) | 61.2 (2.6) | 61.8 (2.9) | 0.002 |
| Body mass index, kg/m², mean (SD) | 27.0 (3.6) | 27.7 (4.2) | 0.013 |
| Educational status, n (%) |  |  | <0.001 |
| Primary or less | 54 (16.5) | 168 (32.1) | — |
| Upper secondary | 62 (19.0) | 133 (25.4) | — |
| Lower tertiary | 104 (31.8) | 129 (24.7) | — |
| Upper tertiary | 107 (32.7) | 93 (17.8) | — |
| Smoking status, n (%) |  |  | <0.001 |
| Never | 95 (29.1) | 131 (25.0) | — |
| Former | 175 (53.5) | 221 (42.3) | — |
| Light | 17 (5.2) | 34 (6.5) | — |
| Regular | 40 (12.2) | 137 (26.2) | — |
| Alcohol use, n (%) |  |  | 0.009 |
| None | 15 (4.6) | 48 (9.2) | — |
| Light | 78 (23.9) | 147 (28.1) | — |
| Regular | 234 (71.6) | 328 (62.7) | — |
| Chronic diseases, n (%) |  |  | <0.001 |
| None | 195 (59.6) | 225 (43.0) | — |
| 1 disease | 102 (31.2) | 181 (34.6) | — |
| ≥2 diseases | 30 (9.2) | 117 (22.4) | — |
| Diabetes at baseline, yes n (%) | 40 (12.2) | 118 (22.6) | <0.001 |
| LTPA, METh/wk, mean (SD) | 46.8 (34.9) | 44.7 (41.2) | 0.440 |
| LTPA category, n (%) |  |  | 0.002 |
| Low (<16.7 METh/wk) | 45 (13.8) | 124 (23.7) | — |
| Moderate (16.7-50 METh/wk) | 166 (50.8) | 234 (44.7) | — |
| High (≥50 METh/wk) | 116 (35.5) | 165 (31.5) | — |
| Dietary intake, mean (SD) |  |  | — |
| Total energy, kJ/day | 10479.8 (3346.5) | 10463.4 (3837.8) | 0.948 |
| Protein, E% | 16.7 (2.3) | 16.7 (2.7) | 0.953 |
| Carbohydrates, E% | 45.6 (6.4) | 45.6 (6.8) | 0.985 |
| Fat, E% | 33.7 (5.3) | 33.7 (5.7) | 0.976 |
| Saturated fat, E% | 12.0 (2.5) | 12.2 (2.5) | 0.201 |
| PUFA, E% | 5.4 (1.2) | 5.2 (1.2) | 0.012 |
| MUFA, E% | 11.4 (2.1) | 11.3 (2.3) | 0.948 |
| Alcohol, E% | 4.3 (4.4) | 4.3 (5.4) | 0.940 |

*Note. p-values derived from independent samples t-tests for continuous variables and chi-square tests for categorical variables.*

*Abbreviations. SD = standard deviation; LTPA = leisure-time physical activity; PUFA = polyunsaturated fatty acids; MUFA = monounsaturated fatty acids.*

**eTable 6. P-values for nominal associations between the volume of leisure-time physical activity and metabolomic modules and biomarkers in late midlife (baseline) in the crude and fully adjusted model in women and men.**

|  | **Women** | | **Men** | |  |
| --- | --- | --- | --- | --- | --- |
| **Module / Biomarker** | **Crude model** | **Fully adjusted model** | **Crude model** | **Fully adjusted model** | |
| LDL-ApoB module | 0.519 | 0.574 | 0.092 | 0.108 | |
| Large VLDL module | 0.878 | 0.380 | 0.023* | 0.181 | |
| HDL-ApoA1 module | 0.205 | 0.128 | 0.200 | 0.009* | |
| Small VLDL module | 0.836 | 0.551 | 0.037* | 0.140 | |
| Large HDL module | 0.534 | 0.536 | 0.403 | 0.703 | |
| TG-FA module | 0.810 | 0.829 | 0.007* | 0.356 | |
| BCAA module | 0.063 | 0.058 | 0.315 | 0.472 | |
| Phospholipid module | 0.329 | 0.244 | 0.165 | 0.073 | |
| Extra-small VLDL module | 0.988 | 0.823 | 0.034* | 0.227 | |
| Small HDL module | 0.168 | 0.066 | 0.350 | 0.007* | |
| HDL-TG module | 0.485 | 0.622 | 0.056 | 0.420 | |
| LDL size | 0.575 | 0.494 | 0.094 | 0.313 | |
| Degree FA of unsaturation | 0.070 | 0.146 | 0.006* | 0.395 | |
| Omega-3 FAs | 0.385 | 0.423 | 0.527 | 0.354 | |
| Docosahexaenoic acid | 0.271 | 0.352 | 0.319 | 0.592 | |
| Alanine | 0.011* | 0.023* | 0.011* | 0.290 | |
| Glutamine | 0.065 | 0.144 | 0.279 | 0.248 | |
| Glycine | 0.812 | 0.944 | 0.460 | 0.180 | |
| Histidine | 0.160 | 0.185 | 0.837 | 0.823 | |
| Phenylalanine | 0.056 | 0.260 | 0.004* | 0.174 | |
| Tyrosine | 0.311 | 0.796 | 0.467 | 0.677 | |
| Glucose | 0.179 | 0.971 | 0.009* | 0.844 | |
| Lactate | 0.059 | 0.417 | 0.004* | 0.340 | |
| Pyruvate | 0.050 | 0.414 | 0.001* | 0.194 | |
| Citrate | 0.026* | 0.021* | 0.218 | 0.496 | |
| Glycerol | 0.080 | 0.939 | 0.000* | 0.098 | |
| 3-hydroxybutyrate | 0.123 | 0.157 | 0.082 | 0.146 | |
| Acetate | 0.037* | 0.159 | 0.246 | 0.203 | |
| Acetoacetate | 0.274 | 0.469 | 0.208 | 0.433 | |
| Acetone | 0.164 | 0.185 | 0.061 | 0.144 | |
| Creatinine | 0.258 | 0.346 | 0.334 | 0.780 | |
| Albumin | 0.001* | 0.006* | 0.148 | 0.331 | |
| GlycA | 0.009* | 0.375 | 0.000* | 0.109 | |

*Note. Crude model: adjusted for age. Fully adjusted model: adjusted for age, body mass index, educational level, alcohol consumption, smoking, diabetes, chronic diseases, and dietary variables including total energy intake and the percentage of proteins, carbohydrates, polyunsaturated fatty acids and monounsaturated fatty acids from total energy intake.*

*Abbreviations: LDL = low-density lipoprotein; ApoB = apolipoprotein B; VLDL = very low-density lipoprotein; HDL = high-density lipoprotein; ApoA1 = apolipoprotein A1; TG = triglyceride; FA = fatty acid; BCAA = branched-chain amino acid; GlycA = glycoprotein acetyls.*

**<.05*

**eTable 7.** **Q-values (multiple-testing corrected p-values) for associations between the volume of leisure-time physical activity and metabolomic modules and biomarkers in late midlife (baseline) in the crude and fully adjusted model in women and men.**

|  | **Women** | | **Men** | |  |
| --- | --- | --- | --- | --- | --- |
| **Module / Biomarker** | **Crude model** | **Fully adjusted model** | **Crude model** | **Fully adjusted model** | |
| LDL-ApoB module | 0.653 | 0.728 | 0.182 | 0.478 | |
| Large VLDL module | 0.905 | 0.665 | 0.075 | 0.478 | |
| HDL-ApoA1 module | 0.377 | 0.510 | 0.328 | 0.153 | |
| Small VLDL module | 0.890 | 0.727 | 0.101 | 0.478 | |
| Large HDL module | 0.653 | 0.727 | 0.459 | 0.773 | |
| TG-FA module | 0.890 | 0.912 | 0.033* | 0.535 | |
| BCAA module | 0.211 | 0.434 | 0.405 | 0.599 | |
| Phospholipid module | 0.473 | 0.612 | 0.287 | 0.478 | |
| Extra-small VLDL module | 0.988 | 0.912 | 0.101 | 0.500 | |
| Small HDL module | 0.346 | 0.434 | 0.412 | 0.153 | |
| HDL-TG module | 0.641 | 0.760 | 0.143 | 0.571 | |
| LDL size | 0.678 | 0.709 | 0.182 | 0.535 | |
| Degree FA of unsaturation | 0.211 | 0.510 | 0.033* | 0.566 | |
| Omega-3 FAs | 0.529 | 0.665 | 0.544 | 0.535 | |
| Docosahexaenoic acid | 0.431 | 0.665 | 0.405 | 0.698 | |
| Alanine | 0.118 | 0.254 | 0.041* | 0.535 | |
| Glutamine | 0.211 | 0.510 | 0.383 | 0.512 | |
| Glycine | 0.890 | 0.971 | 0.497 | 0.478 | |
| Histidine | 0.346 | 0.510 | 0.837 | 0.844 | |
| Phenylalanine | 0.211 | 0.612 | 0.030* | 0.478 | |
| Tyrosine | 0.466 | 0.912 | 0.497 | 0.771 | |
| Glucose | 0.348 | 0.971 | 0.038* | 0.844 | |
| Lactate | 0.211 | 0.665 | 0.030* | 0.535 | |
| Pyruvate | 0.211 | 0.665 | 0.010* | 0.478 | |
| Citrate | 0.211 | 0.254 | 0.328 | 0.606 | |
| Glycerol | 0.221 | 0.971 | 0.003* | 0.478 | |
| 3-hydroxybutyrate | 0.311 | 0.510 | 0.181 | 0.478 | |
| Acetate | 0.211 | 0.510 | 0.353 | 0.478 | |
| Acetoacetate | 0.431 | 0.704 | 0.328 | 0.571 | |
| Acetone | 0.346 | 0.510 | 0.144 | 0.478 | |
| Creatinine | 0.431 | 0.665 | 0.408 | 0.830 | |
| Albumin | 0.040* | 0.198 | 0.272 | 0.535 | |
| GlycA | 0.118 | 0.665 | 0.003* | 0.478 | |

*Note. Crude model: adjusted for age. Fully adjusted model: adjusted for age, body mass index, educational level, alcohol consumption, smoking, diabetes, chronic diseases, and dietary variables including total energy intake and the percentage of proteins, carbohydrates, polyunsaturated fatty acids and monounsaturated fatty acids from total energy intake.*

*Abbreviations: LDL = low-density lipoprotein; ApoB = apolipoprotein B; VLDL = very low-density lipoprotein; HDL = high-density lipoprotein; ApoA1 = apolipoprotein A1; TG = triglycerides; FA = fatty acid; BCAA = branched-chain amino acid; GlycA = glycoprotein acetyls.*

**<.05*

**eTable 8.** **Age x Leisure-time physical activity interaction p-values for longitudinal metabolomic trajectories of modules and biomarkers in women and men before (p-value) and after (q-value) correcting for multiple testing.**

|  | **Women** | | **Men** | |
| --- | --- | --- | --- | --- |
| **Module / Biomarker** | **p-value** | **q-value** | **p-value** | **q-value** |
| LDL-ApoB module | 0.361 | 0.825 | 0.902 | 0.968 |
| Large VLDL module | 0.896 | 0.954 | 0.006* | 0.094 |
| HDL-ApoA1 module | 0.404 | 0.825 | 0.899 | 0.968 |
| Small VLDL module | 0.410 | 0.825 | 0.096 | 0.599 |
| Large HDL module | 0.685 | 0.938 | 0.704 | 0.968 |
| TG-FA module | 0.966 | 0.966 | 0.034* | 0.274 |
| BCAA module | 0.109 | 0.825 | 0.017* | 0.183 |
| Phospholipid module | 0.481 | 0.825 | 0.580 | 0.939 |
| Extra-small VLDL module | 0.649 | 0.938 | 0.522 | 0.939 |
| Small HDL module | 0.424 | 0.825 | 0.962 | 0.968 |
| HDL-TG module | 0.723 | 0.938 | 0.001* | 0.041* |
| LDL size | 0.811 | 0.938 | 0.429 | 0.939 |
| Degree FA of unsaturation | 0.500 | 0.825 | 0.112 | 0.599 |
| Omega-3 FAs | 0.206 | 0.825 | 0.471 | 0.939 |
| Docosahexaenoic acid | 0.271 | 0.825 | 0.791 | 0.968 |
| Alanine | 0.318 | 0.825 | 0.594 | 0.939 |
| Glutamine | 0.461 | 0.825 | 0.157 | 0.626 |
| Glycine | 0.231 | 0.825 | 0.271 | 0.867 |
| Histidine | 0.364 | 0.825 | 0.209 | 0.742 |
| Phenylalanine | 0.853 | 0.938 | 0.852 | 0.968 |
| Tyrosine | 0.841 | 0.938 | 0.968 | 0.968 |
| Glucose | 0.424 | 0.825 | 0.908 | 0.968 |
| Lactate | 0.220 | 0.825 | 0.510 | 0.939 |
| Pyruvate | 0.706 | 0.938 | - | - |
| Citrate | 0.966 | 0.966 | 0.579 | 0.939 |
| Glycerol | 0.298 | 0.825 | 0.475 | 0.939 |
| 3-hydroxybutyrate | 0.445 | 0.825 | 0.616 | 0.939 |
| Acetate | 0.292 | 0.825 | 0.504 | 0.939 |
| Acetoacetate | 0.545 | 0.856 | 0.896 | 0.968 |
| Acetone | 0.207 | 0.825 | 0.805 | 0.968 |
| Creatinine | 0.197 | 0.825 | 0.147 | 0.626 |
| Albumin | 0.819 | 0.938 | 0.465 | 0.939 |
| GlycA | 0.750 | 0.938 | 0.765 | 0.968 |

*Note. (-) = These models could not be calculated due to convergence issues.*

*Abbreviations: LDL = low-density lipoprotein; ApoB = apolipoprotein B; VLDL = very low-density lipoprotein; HDL = high-density lipoprotein; ApoA1 = apolipoprotein A1; TG = triglycerides; FA = fatty acid; BCAA = branched-chain amino acid; GlycA = glycoprotein acetyls.*

**<.05*

**eTable 9. Pairwise contrasts between low, moderate and high leisure-time physical activity (LTPA) groups in men for metabolomic biomarker modules with a nominally significant age × LTPA interaction (p<0.05), showing uncorrected p-values and multiple-testing-corrected q-values.**

|  | **Low vs moderate** | | **High vs moderate** | | **Low vs high** | |
| --- | --- | --- | --- | --- | --- | --- |
| **Module / Biomarker** | **p-value** | **q-value** | **p-value** | **q-value** | **p-value** | **q-value** |
| Large VLDL module | 0.083 | 0.110 | 0.140 | 0.168 | 0.001* | 0.004* |
| TG-FA module | 0.172 | 0.188 | 0.075 | 0.110 | 0.010* | 0.023* |
| BCAA module | 0.009* | 0.023* | 0.278 | 0.278 | 0.001* | 0.004* |
| HDL-TG module | 0.077 | 0.110 | 0.022* | 0.045* | 0.001* | 0.004* |

*Abbreviations: VLDL = very low-density lipoprotein; HDL = high-density lipoprotein; TG = triglyceride; FA = fatty acid; BCAA = branched-chain amino acid.*

**<.05*

**eTable 10.** **Age x Leisure-time physical activity change interaction p-values for longitudinal metabolomic trajectories of modules and biomarkers in women and men before (p-value) and after (q-value) correcting for multiple testing.**

|  | **Women** | | **Men** | |
| --- | --- | --- | --- | --- |
| **Module / Biomarker** | **p-value** | **q-value** | **p-value** | **q-value** |
| LDL-ApoB module | 0.990 | 0.990 | 0.943 | 0.973 |
| Large VLDL module | 0.719 | 0.989 | 0.571 | 0.973 |
| HDL-ApoA1 module | 0.701 | 0.989 | 0.758 | 0.973 |
| Small VLDL module | 0.768 | 0.990 | 0.756 | 0.973 |
| Large HDL module | 0.138 | 0.822 | 0.991 | 0.991 |
| TG-FA module | 0.914 | 0.990 | 0.423 | 0.973 |
| BCAA module | 0.024* | 0.404 | 0.205 | 0.973 |
| Phospholipid module | 0.957 | 0.990 | 0.803 | 0.973 |
| Extra-small VLDL module | 0.986 | 0.990 | 0.802 | 0.973 |
| Small HDL module | 0.321 | 0.928 | 0.620 | 0.973 |
| HDL-TG module | 0.887 | 0.990 | 0.186 | 0.973 |
| LDL size | 0.270 | 0.928 | 0.460 | 0.973 |
| Degree FA of unsaturation | 0.814 | 0.990 | 0.212 | 0.973 |
| Omega-3 FAs | 0.506 | 0.928 | 0.850 | 0.973 |
| Docosahexaenoic acid | 0.426 | 0.928 | 0.921 | 0.973 |
| Alanine | 0.174 | 0.822 | 0.508 | 0.973 |
| Glutamine | 0.594 | 0.989 | 0.709 | 0.973 |
| Glycine | 0.350 | 0.928 | 0.380 | 0.973 |
| Histidine | 0.043* | 0.470 | 0.717 | 0.973 |
| Phenylalanine | 0.078 | 0.645 | 0.729 | 0.973 |
| Tyrosine | 0.157 | 0.822 | 0.673 | 0.973 |
| Glucose | 0.612 | 0.989 | 0.851 | 0.973 |
| Lactate | 0.650 | 0.989 | 0.296 | 0.973 |
| Pyruvate | 0.425 | 0.928 | 0.414 | 0.973 |
| Citrate | 0.478 | 0.928 | - | - |
| Glycerol | 0.697 | 0.989 | 0.742 | 0.973 |
| 3-hydroxybutyrate | 0.283 | 0.928 | 0.608 | 0.973 |
| Acetate | 0.470 | 0.928 | 0.758 | 0.973 |
| Acetoacetate | 0.336 | 0.928 | 0.445 | 0.973 |
| Acetone | 0.506 | 0.928 | 0.614 | 0.973 |
| Creatinine | 0.024* | 0.404 | 0.907 | 0.973 |
| Albumin | 0.800 | 0.990 | 0.219 | 0.973 |
| GlycA | 0.859 | 0.990 | 0.528 | 0.973 |

*Note. (-) = These models could not be calculated due to convergence issues.*

*Abbreviations:LDL = low-density lipoprotein; ApoB = apolipoprotein B; VLDL = very low-density lipoprotein; HDL = high-density lipoprotein; ApoA1 = apolipoprotein A1; TG = triglycerides; FA = fatty acid; BCAA = branched-chain amino acid; GlycA = glycoprotein acetyls.*

**<.05*
